# Unveiling the link between oral microbial α-diversity, advanced cardiovascular-kidney-metabolic syndrome, and mortality

**DOI:** 10.1080/20002297.2026.2717777

**Published:** 2026-08-21

**Authors:** Zunni Zhang, Huiying Ji, Yumei Xu, Yunxiao Song, Jie Chen

**Affiliations:** a Department of Laboratory Medicine, Shanghai Xuhui District Central Hospital & Zhongshan-Xuhui Hospital, Fudan University, Shanghai, People's Republic of China

**Keywords:** α-diversity, cardiovascular-kidney-metabolic syndrome, frailty, mortality, oral microbiome

## Abstract

**Background:**

Prior studies have linked oral microbial diversity to separate cardiometabolic endpoints, yet none have applied the cardiovascular-kidney-metabolic (CKM) framework.

**Objective:**

This study aimed to evaluate the association between oral microbiome *α*-diversity and advanced CKM, mortality, and potential mediators.

**Design:**

We analyzed 6,044 adults (≥30 years) from NHANES 2009–2012. 402 all-cause deaths (144 CVD deaths) were recorded. Four *α*-diversity metrics (observed amplicon sequence variants (ASVs), Faith's PD, Shannon–Weiner index, Simpson index) from 16S rRNA sequencing of oral rinse samples were used to assess associations with advanced CKM (logistic regression) and all-cause/CVD mortality (Cox regression). Restricted cubic splines (RCS) and generalized additive models (GAM) characterized dose–response shapes. The findings were verified through four sets of sensitivity analyses. Mediation analyses examined two candidate mediators—a 49-item frailty index and the systemic immune-inflammation index (SII).

**Results:**

Higher *α*-diversity was inversely associated with advanced CKM (strongest for Faith's PD, per 1-SD: OR = 0.91, 95% CI 0.86–0.98, Q4 vs Q1 OR = 0.70, 95% CI 0.58–0.84, *p*-trend < 0.001). All four *α*-diversity metrics were inversely associated with all-cause mortality, with Observed ASVs and Faith's PD exhibiting L-shaped dose-response patterns (*p*-nonlinear = 0.017 and 0.012, respectively), the Shannon–Weiner index exhibited a strictly linear inverse association (*p*-nonlinear = 0.445; EDF = 1.00), and the Simpson index showed a near-linear pattern (*p*-nonlinear = 0.05, *p*-overall < 0.001, EDF = 1.92). For CVD mortality, Shannon–Weiner index (per 1-SD: HR = 0.79, 95% CI 0.68–0.92, Q4 vs Q1 HR = 0.62, 95% CI 0.39–1.00, *p*-trend = 0.018) and Simpson index (HR = 0.82, 95% CI 0.72–0.93, Q4 vs Q1 HR = 0.57, 95% CI 0.35–0.93, *p*-trend = 0.024) maintained the most robust associations. Findings were robust across four sensitivity analyses. Exploratory mediation analyses estimated that the indirect effect of frailty on mortality was 14.30%–37.20%.

**Conclusion:**

This study suggests that oral microbiome *α*-diversity may be associated with CKM risk, and frailty may represent a potential mediator.

## Graphical abstract

**Figure:**
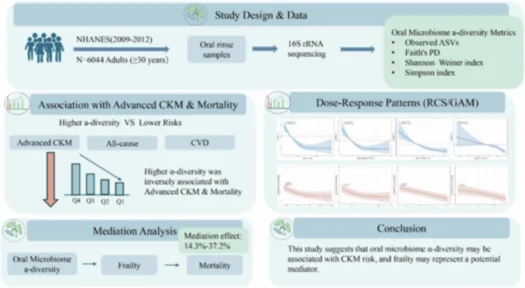

## Introduction

The human oral cavity harbours the second most diverse microbial ecosystem in the body, after the gut [1]. Oral microbial communities are sustained by complex cooperative and antagonistic interspecies interactions, and their composition has emerging implications for both local and systemic health [2]. Modulation of host microbial ecosystems to potentiate immune responses and attenuate chronic disease has become an active translational research frontier [3]. However, mechanistic and epidemiological evidence on the oral microbiome remains far less developed than that on the gut microbiome [4], even as accumulating data link oral dysbiosis with cardiometabolic pathology [5–7] and a broad spectrum of systemic conditions, including malignancy, CVD, and diabetes [8,9].

In October 2023, the American Heart Association (AHA) introduced the concept of cardiovascular-kidney-metabolism syndrome (CKM), a progressive, systemic health condition characterised by the concurrent presence of obesity, diabetes mellitus (DM), chronic kidney disease, and cardiovascular disease (CVD) [10]. CKM syndrome reflects shared pathophysiological pathways across these traditionally siloed conditions and substantially elevates the risk of adverse cardiovascular events and premature mortality [11]. More than 25% of US adults are estimated to meet criteria for CKM syndrome, and over 75% of cardiometabolic-related health expenditures in the United States are devoted to managing affected individuals [12].

Several critical gaps remain. First, although prior NHANES analyses have linked lower oral microbial *α*-diversity to higher all-cause and CVD mortality [13], and other studies have examined oral microbial diversity in relation to isolated cardiovascular or metabolic endpoints [14,15], no investigation to date has adopted the AHA CKM staging system—a unifying framework that captures the progressive, interconnected nature of metabolic, renal, and cardiovascular dysfunction—as a primary outcome, nor systematically compared multiple *α*-diversity indices across this spectrum. Whether oral microbial *α*-diversity is differentially associated with CKM stage advancement and how it relates to subsequent all-cause and CVD mortality within an integrated cardiometabolic framework remains unestablished. Second, the biological pathways through which oral microbial communities may influence cardiometabolic risk are poorly characterised. The SII, an integrated marker reflecting peripheral immune cell dynamics [16], and frailty, a multidimensional indicator of physiological vulnerability [17], were evaluated as two candidate statistical mediators that may reflect different dimensions of systemic vulnerability; however, their causal or mechanistic roles remain uncertain. Third, dose-response relationships, threshold effects, and effect modification by demographic and lifestyle factors—all critical for translational interpretation—have not been systematically characterised for oral microbial *α*-diversity in the context of CKM.

To address these gaps, we aimed to: (1) investigate the association between oral microbial *α*-diversity and CKM stage; (2) examine the relationship between oral microbial *α*-diversity and CKM mortality risk. (3) explore SII and frailty index (FI) as potential mediators between *α*-diversity and CKM mortality.

## Methods

### Study design and data source

Utilising data from two consecutive cycles (2009–2010 and 2011–2012) of the National Health and Nutrition Examination Survey (NHANES), the only cycles in which oral microbiome sequencing was performed. Mortality follow-up was ascertained by the Centres for Disease Control and Prevention website (https://wwwn.cdc.gov/nchs/nhanes/Default.aspx) through December 31, 2019. NHANES is a continuous, multistage probability survey of the noninstitutionalized civilian US population conducted by the National Centre for Health Statistics (NCHS). The protocol was approved by the NCHS Ethics Review Board, and all participants provided written informed consent. The present analysis followed the Strengthening the Reporting of Observational Studies in Epidemiology (STROBE) guidelines.

### Study population

**Figure 1.**
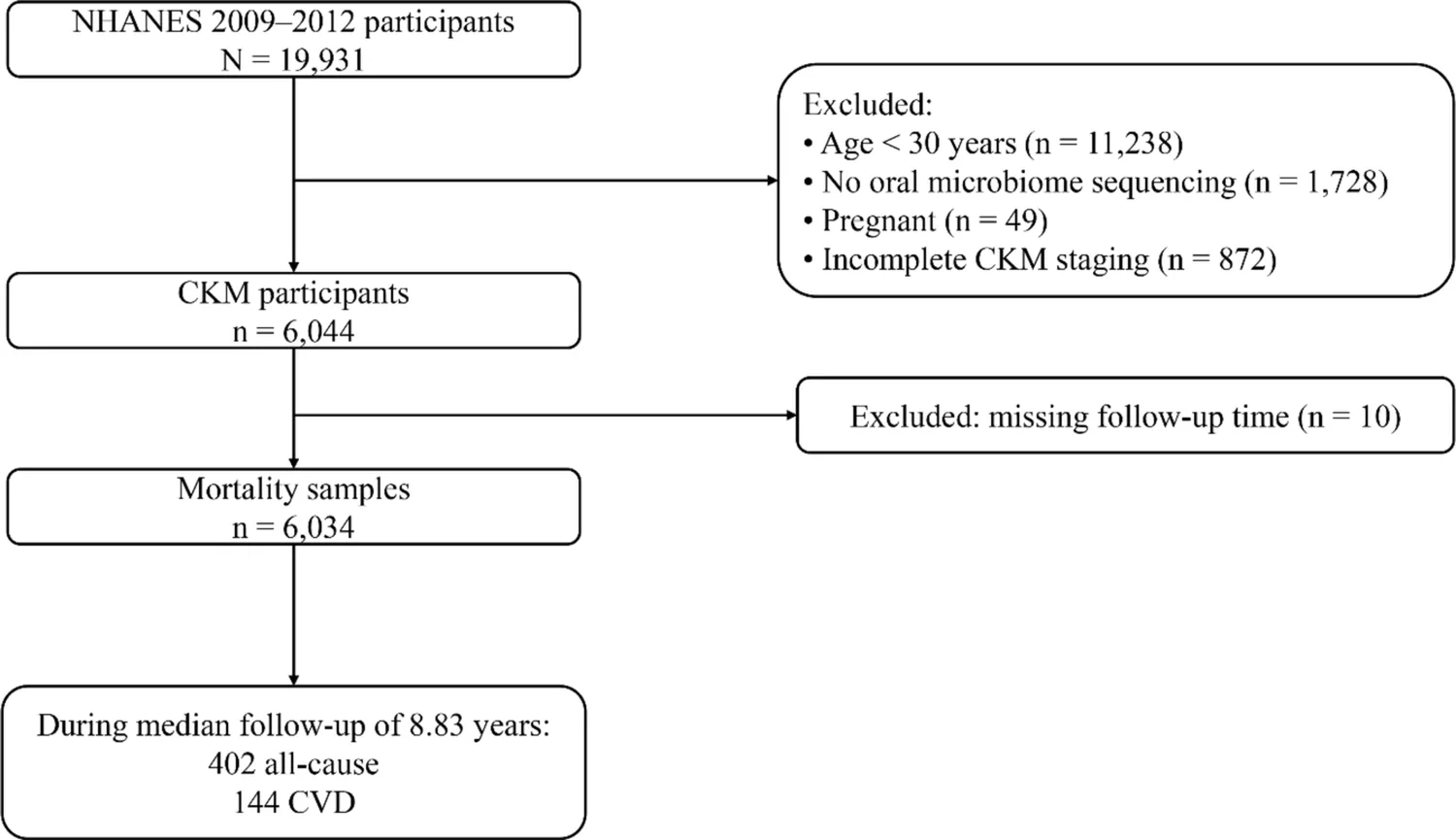
The study flow diagram of this study.

From 19,931 initial participants, we sequentially excluded individuals younger than 30 years (*n* = 11,238), those without oral microbial sequencing data (*n* = 1,728), pregnant women (*n* = 49), and participants with incomplete data on CKM staging (*n* = 872). The final analytic sample comprised 6,044 individuals, with complete follow-up data available for 6,034 participants. The study flow diagram is shown in Figure 1.

### CKM syndrome staging

CKM syndrome was staged in accordance with the AHA 2023 Presidential Advisory [10]. Stage 0: no CKM risk factors. Stage 1: is characterised by excess or dysfunctional adiposity. Stage 2: involves metabolic risk factors or moderate-to-severe risk chronic kidney disease (CKD). Stage 3: defined as the presence of subclinical CVD. Stage 4: characterised by clinical CVD. Subclinical CVD was calculated by the AHA PREVENT equation [18]. The definition of CKM conditions was shown in Table S1, and the CKM stage details were shown in Table S2. CKM was categorised into early (Stage 0–2) and advanced (Stage 3–4) groups [19].

### Oral Microbiome sampling and *α*-diversity quantification

Oral rinse samples, laboratory, and bioinformatic processing were performed centrally under the NHANES Oral Microbiome Project (https://wwwn.cdc.gov/Nchs/Nhanes/Omp/Default.aspx): participants rinsed and gargled with mouthwash for 5 seconds each, repeated three times, and expectorated into a collection cup. DNA was extracted from oral rinse samples using the Pure Gene DNA purification kit, and the 16S rRNA gene was amplified by polymerase chain reaction before sequencing. Sequence reads were processed through DADA2/QIIME to compute *α*-diversity indices [20]. Four metrics (observed ASVs, Faith's phylogenetic diversity (PD), Shannon-Weiner index, Simpson Index) were obtained from the NHANES 2009–2012 Oral Microbiome public-use file dada2rsv-alpha.txt and were calculated at a rarefied depth of 10,000 sequences per sample, for it was the highest uniform depth available for all participants included in the analytic sample, thereby retaining the greatest amount of sequencing information without additional sample exclusion. For each metric, the ten random rarefaction replicates at this depth were averaged to reduce stochastic variation arising from individual subsampling iterations [21]. The observed ASVs and Faith’s PD metric measure richness. The Shannon-Weiner and Simpson index measure richness and evenness [22–24].

### Definitions of covariates

Covariates included age, sex, race/ethnicity (Non-Hispanic White, Non-Hispanic Black, Mexican American, Hispanic, and Other), education level, poverty income ratio (PIR), smoking status (ever smoked ≥ 100 cigarettes), alcohol consumption (ever had ≥ 12 drinks in lifetime), marital status and periodontitis (one or more sites with clinical attachment loss ≥ 3 mm and probing depth ≥ 4 mm following the CDC/AAP 2007 criteria [25]). Systemic immune-inflammation index (SII = platelet count × neutrophil count/lymphocyte count) [26]. A 49-item FI (Table S3) was constructed based on the cumulative deficit model [27], adapted from the framework for the NHANES population [28,29]. For categorical analyses, participants were classified as robust (FI ≤ 0.10), pre-frail (0.10 < FI < 0.25), or frail (FI ≥ 0.25) [28,30].

### Missing data handling

Missingness across covariates ranged from 0% to 12.52% (periodontitis) (Table S4), with an overall missing-completely-at-random pattern (Little's MCAR test *P* = 0.18). Multiple imputation by chained equations (MICE) was performed with five imputed datasets (m = 5, 50 iterations), using predictive mean matching for continuous variables and polytomous logistic regression for categorical variables. Pooled estimates were obtained using Rubin's rules across imputed datasets.

### Mortality outcomes

Mortality follow-up data were obtained from the Centres for Disease Control and Prevention website (https://wwwn.cdc.gov/nchs/nhanes/Default.aspx) through December 31, 2019. The primary outcomes were all-cause mortality and CVD mortality identified using ICD-10 codes.

### Statistical analysis

#### Primary regression analyses

All models were fitted without incorporating NHANES survey weights, strata, or primary sampling units. *α*-Diversity metrics were modelled in five complementary forms in primary regression analyses: (i) as a continuous variable standardised to z-scores (per 1-SD increment), computed within each analytical sample to ensure internal scale consistency; (ii–iv) quartiles (Q1–Q4), with cut points derived independently within each analytical sample (advanced CKM sample, *n* = 6,044; mortality sample, *n* = 6,034) and reported in Table S5; and (v) test for trend across quartiles. The same z-score transformation was applied across primary regression, subgroup, sensitivity, and mediation analyses to ensure that effect estimates are interpreted in a unified per-1 SD manner. Restricted cubic spline (RCS) and generalised additive model (GAM) analyses retained the original (non-standardised) *α*-diversity scale to preserve clinical interpretability of dose-response thresholds.

The proportional hazards assumption was verified for each exposure–outcome model using scaled Schoenfeld residuals, evaluated for the *α*-diversity exposure, for every covariate individually, and by a global test; no violations were detected (all *P* > 0.05; Table S6). For advanced CKM, multivariable logistic regression yielded odds ratios (OR) with 95% confidence intervals (CIs). For all-cause and CVD mortality, multivariable Cox proportional hazards regression yielded hazard ratios (HR) with 95% CI. In the CVD mortality Cox models, deaths from non-cardiovascular causes were treated as censoring events at the date of death. Three adjusted models were fitted for each outcome–metric combination: Model 1 adjusted for age and sex; Model 2 additionally adjusted for race/ethnicity, education, smoking, alcohol, marital status, and PIR; Model 3 further adjusted for periodontitis. The *P* values of the main analysis were corrected by the Benjamini-Hochberg FDR.

#### Multicollinearity assessment

Multicollinearity among covariates was assessed using the generalised variance inflation factor (GVIF) and its scaled form, GVIF^(1/(2·df)), interpreted as: <2, negligible; 2.00–2.24, mild; 2.24–3.16, moderate; >3.16, severe collinearity. All covariates exhibited GVIF^(1/(2·df)) values between 1.04 and 1.19, indicating negligible collinearity. Because each *α*-diversity metric was modelled independently rather than jointly, multicollinearity among diversity indices was not a source of bias for the primary effect estimates (Table S7).

#### Restricted Cubic Spline (RCS) and Generalised Additive Model (GAM) analyses

RCS analyses were used to characterise potential non-linear dose-response relationships between *α*-diversity and outcomes. Reference values were set at the 5th percentile of each metric, anchoring all OR/HR estimates relative to the lowest-diversity stratum. To determine the optimal number of knots, the Akaike Information Criterion (AIC) and the Bayesian Information Criterion (BIC) were computed across 3-, 4-, 5-, and 6-knot configurations. The BIC, which more strongly penalises overfitting, was prioritised for final selection. The results of AIC and BIC, as well as the final determined number of knots for each metric–outcome combination, are reported in Table S8. GAM analyses with thin-plate regression splines were used as a complementary check on linearity, with effective degrees of freedom (EDF) interpreted as: EDF = 1, linear; 1 < EDF < 3, mild curvilinearity; EDF ≥ 3, marked non-linearity.

#### Subgroup analyses

Stratified analyses across three prespecified subgroups—sex, age (<65 vs ≥65 years), and race/ethnicity—tested the consistency of *α*-diversity–outcome associations. Two complementary forms of multiplicative interaction were tested: (i) a continuous-by-subgroup product term, reflecting heterogeneity in dose-response slopes, and (ii) a quartile-by-subgroup product term, reflecting heterogeneity in extreme group contrasts.

#### Mediation analyses

We conducted exploratory mediation analyses using SII and FI as two candidate statistical mediators. These analyses were intended to characterise indirect associations. For logistic outcomes, the ‘mediation’ R package was used with bias-corrected and accelerated (BCa) bootstrap confidence intervals (1,000 resamples). For Cox outcomes, manual bootstrap-based mediation using the coefficient-difference method was performed (1,000 bootstrap resamples; percentile confidence intervals).

#### Sensitivity analyses

Four sensitivity analyses assessed the robustness of findings: (i) Complete case analysis (CCA) (CKM analyses: *n* = 4,456; mortality analyses: *n* = 4,442) to evaluate whether observed associations might be artifacts of imputation procedures; (ii) Exclusion of participants with prior cancer history (advanced CKM: *n* = 5,631; mortality: *n* = 5,621) to address potential confounding from cancer- or treatment-related shifts in the oral microbiome; and (iii) excluding deaths within the first two years of follow-up (advanced CKM: *n* = 5,980; mortality: *n* = 5,970) to mitigate reverse causation from preclinical disease; and (iv) competing-risk analysis for CVD mortality using the Fine–Grey subdistribution hazard model, in which non-CVD deaths were modelled as a competing, to confirm that the cause-specific estimates were not affected by informative censoring.

### Statistical analysis

Continuous variables were described as mean and standard deviation (SD). Categorical variables were described by counts and percentages. All analyses were performed using R version 4.3.1. Two-sided *P* < 0.05 was considered statistically significant.

## Results

### Baseline characteristics

Among 6,044 participants, the mean (SD) age was 48.87 (11.30) years, and 49.6% (*n* = 2,998) were male. The cohort was predominantly composed of non-Hispanic White (38.6%) and non-Hispanic Black (23.4%) individuals. Ever-smoking was reported by 45.6%, ever-alcohol consumption by 74.1%, and cancer history by 6.8%. The majority of participants were married or living with a partner (64.6%), and 52.4% had attained college-level education or above (Table 1).

**Table 1.** Baseline characteristics of CKM participants.

	level	Overall
n		6044
Age (mean (SD))		48.87 (11.30)
Sex (%)	Female	3046 (50.4)
	Male	2998 (49.6)
Eth (%)	Non-Hispanic White	2332 (38.6)
	Non-Hispanic Black	1415 (23.4)
	Mexican American	988 (16.3)
	Hispanic and Other	1309 (21.7)
Education (%)	Less than high school	1577 (26.1)
	High school	1301 (21.5)
	College or above	3166 (52.4)
PIR (%)	Low	2012 (33.3)
	Intermediate	1716 (28.4)
	High	2316 (38.3)
Marital (%)	Married/Living with partner	3906 (64.6)
	Separated/Divorced/Widowed	1358 (22.5)
	Never married	780 (12.9)
Smoking (%)	No	3286 (54.4)
	Yes	2758 (45.6)
Alcohol (%)	No	1563 (25.9)
	Yes	4481 (74.1)
Cancer (%)	No	5631 (93.2)
	Yes	413 (6.8)
Periodontitis (%)	No	3292 (54.5)
	Yes	2752 (45.5)
Observed ASVs (mean (SD))		129.58 (45.05)
Faith’s PD (mean (SD))		14.72 (3.86)
Shannon–Weiner index (mean (SD))		4.60 (0.71)
Simpson index (mean (SD))		0.90 (0.06)

During a median follow-up of 8.83 years, 402 all-cause deaths were recorded, of which 144 (35.8%) were attributed to CVD. The event counts across diversity categories are shown in Table S9.

### *α*-diversity and advanced CKM

**Figure 2.**
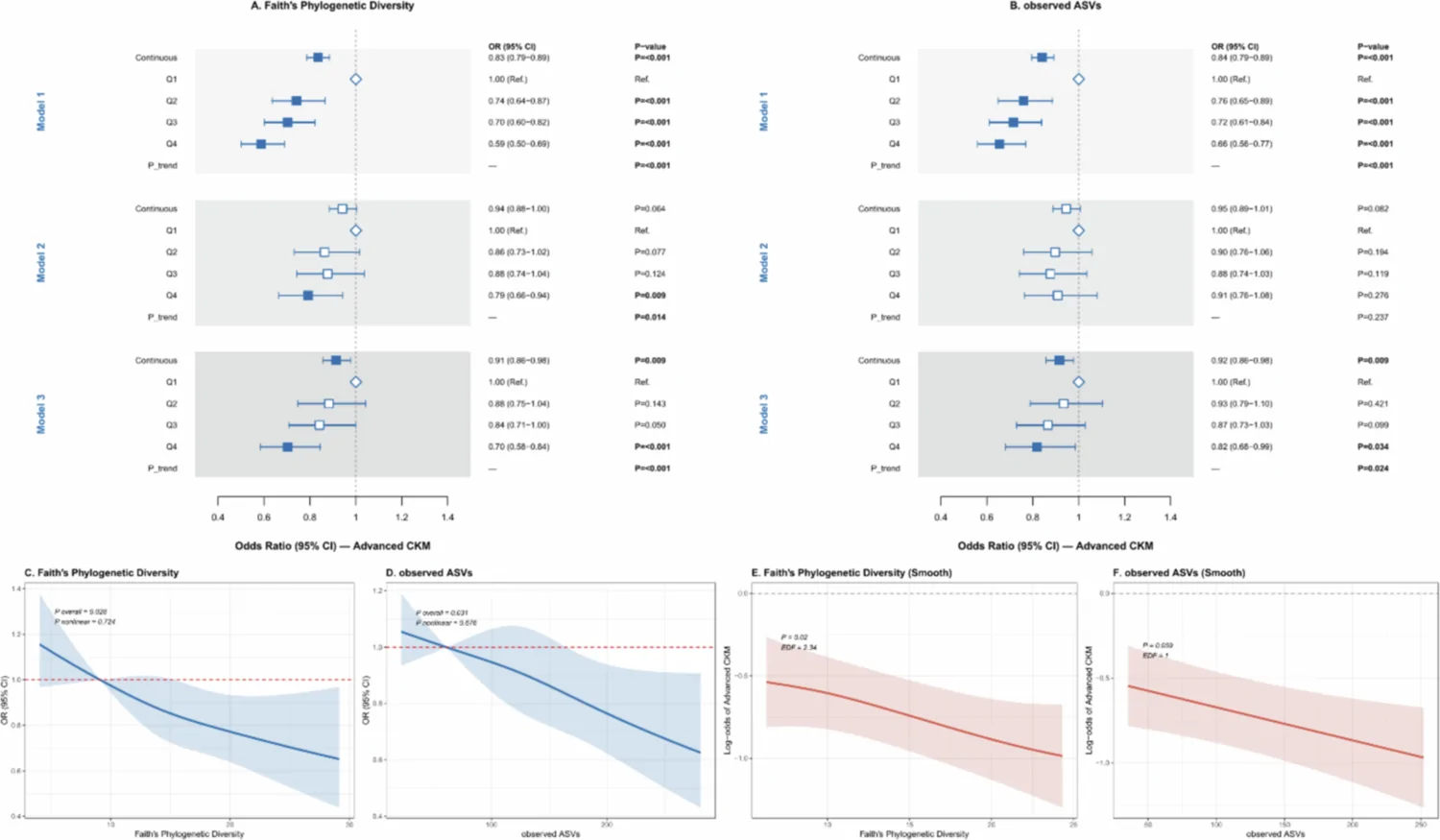
Association between Faith's PD, Observed ASVs, and advanced CKM patients. Forest map (A, B), RCS analysis (C, D), generalised additive model analysis (E, F).

In fully adjusted logistic regression (Model 3), Faith's PD demonstrated the strongest inverse association with advanced CKM, with each 1-SD increment was associated with 9.0% lower odds of advanced CKM (OR = 0.91, 95% CI 0.86–0.98; *P* = 0.009), and the highest versus lowest quartile contrast yielded (OR = 0.70, 95% CI 0.58–0.84; *P*-trend < 0.001) (Figure 2A). Observed ASVs showed similarly directed, but weaker patterns (Q4 vs Q1 OR = 0.82, 95% CI 0.68–0.99; *P* = 0.034; *P*-trend = 0.024); the per 1-SD OR was 0.92(95% CI 0.86–0.98, *P* = 0.009) (Figure 2B). The Shannon–Weiner index and Simpson index did not reach statistical significance (Figure S1A,B).

RCS analyses (Figure 2C,D) showed that both Faith's PD (*P* overall = 0.028) and Observed ASVs (*P* overall = 0.031) demonstrated significant linear inverse associations with advanced CKM (both *P*-nonlinear > 0.6, indicating no evidence of departure from linearity). GAM analysis (Figure 2E,F) was concordant: Faith's PD (*P* = 0.02, EDF = 2.34), Observed ASVs (*P* = 0.009, EDF = 1).

### *α*-diversity and all-cause mortality

**Figure 3.**
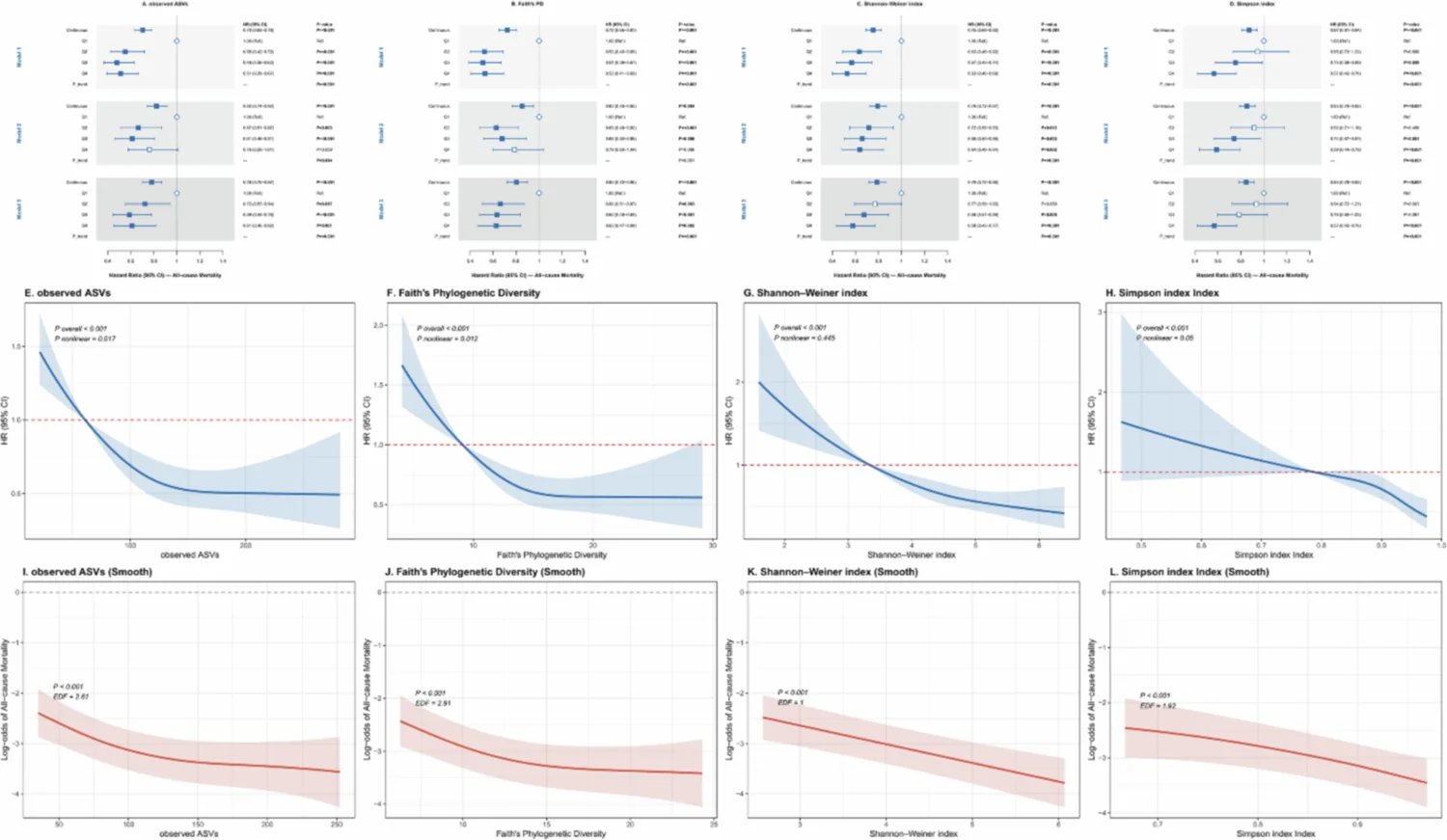
Association between four *α*-diversity metrics and all-cause mortality. Forest map (A–D), RCS analysis (E–H), generalised additive model analysis (I–L).

In fully adjusted Cox regression (Model 3), all four *α*-diversity metrics demonstrated robust inverse associations with all-cause mortality (all *P*-trend < 0.001). Per 1-SD increments yielded HRs of 0.78 (Observed ASVs, 95% CI 0.70–0.87), 0.80 (Faith's PD, 95% CI 0.72–0.90), 0.79 (Shannon–Weiner index, 95% CI 0.72–0.86), and 0.85 (Simpson index, 95% CI 0.78–0.92). Quartile-based contrasts mirrored these patterns: relative to Q1, the highest quartiles (Q4) were associated with HRs of 0.61 for Observed ASVs (*P* = 0.001), 0.63 for Faith's PD (*P* = 0.002), 0.58 for Shannon–Weiner index (*P* < 0.001), and 0.57 for Simpson index (*P* < 0.001) (Figure 3A–D).

RCS analyses revealed two distinct dose-response patterns (Figure 3E–H). Observed ASVs and Faith's PD exhibited L-shaped (threshold) relationships with all-cause mortality (*P* nonlinear = 0.017 and 0.012, respectively): HR estimates declined steeply from approximately 1.5 to 0.5 across the lower-diversity range and plateaued near 0.5 above threshold values of approximately 130 (Observed ASVs) and 14 (Faith's PD). Shannon–Weiner index exhibited a strictly linear inverse association (*P* nonlinear = 0.45; EDF = 1.00), whereas Simpson index showed borderline non-linearity (*P* nonlinear = 0.05) consistent with a near-linear pattern (*p* overall < 0.001, EDF = 1.92).GAM analyses (Figure 3I–L) supported these conclusions.

### *α*-diversity and CVD mortality

**Figure 4.**
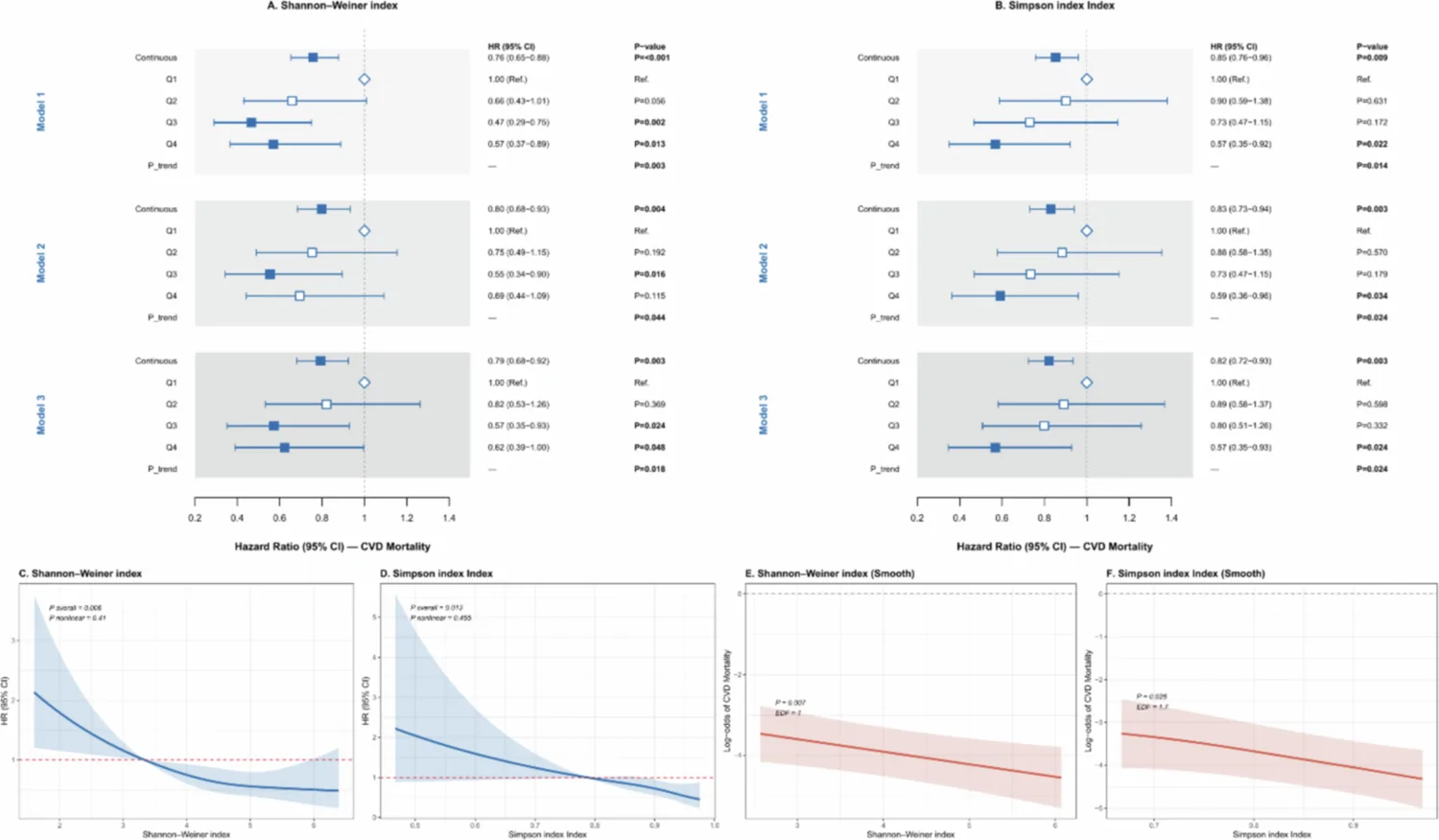
Association between Shannon–Weiner index, Simpson index, and CVD mortality. Forest map (A, B), RCS analysis (C, D), generalised additive model analysis (E, F).

Associations with CVD mortality were attenuated relative to all-cause mortality, but Shannon–Weiner index and Simpson index retained the most robust signals. In Model 3, the per 1-SD HRs were 0.79 (Shannon–Weiner index, 95% CI 0.68–0.92; *P* = 0.003) and 0.82 (Simpson index, 0.72–0.93; *P* = 0.003), with corresponding Q4 vs Q1 HRs of 0.62 and 0.57 (both *P*-trend < 0.05) (Figure 4A,B). Observed ASVs showed a marginal continuous-scale association (per 1-SD: HR = 0.81; *P* = 0.016) that did not reach significance in the Q4 vs Q1 contrast (HR = 0.70; *P* = 0.155). Faith's PD was not significantly associated with CVD mortality after full adjustment (Figure S2A,B).

RCS and GAM analysis confirmed these patterns: Shannon–Weiner index (*P* overall = 0.006, EDF = 1) and Simpson index (*P* overall = 0.013, EDF = 1.7) exhibited approximately linear inverse associations with CVD mortality (both *P* nonlinear > 0.4) (Figure 4C–F). Observed ASVs showed a marginal linear association (*P* overall = 0.043), and Faith's PD displayed no significant overall association (Figure S2C,D).

After Benjamini–Hochberg FDR adjustment across the primary metric–outcome associations (Table S10), all significant metric associations in model 3 remained highly significant (FDR *q* < 0.05).

### Subgroup analyses

Stratified analyses revealed largely consistent associations across sex, age, and race/ethnicity strata. The most notable heterogeneity was a significant race-by-Observed ASVs interaction for advanced CKM (*P*-interaction = 0.04), with a stronger inverse association observed in non-Hispanic Black participants (OR = 0.85; 95% CI 0.75-0.95, *P* = 0.01) (Table S11). The association of *α*-diversity with mortality is generally consistent across the examined subgroups (Tables S12 and 13).

### Sensitivity analyses

Four sensitivity analyses confirmed the robustness of primary findings: First, complete case analysis (CCA: CKM *n* = 4,456; mortality *n* = 4,442) yielded effect estimates highly consistent with—or marginally stronger than—those from multiple imputations, indicating that the observed associations were not artifacts of imputation procedures (Table S14). Second, exclusion of participants with cancer did not materially alter primary findings, arguing against confounding by cancer-related microbiome alterations or treatment effects (Table S15). Third, excluding deaths within the first two years of follow-up, mainly findings were retained, notably (Table S16). Fourth, in competing-risk analyses treating non-CVD death as a competing event, Fine–Grey subdistribution hazard ratios were nearly identical to the corresponding multivariable COX estimates (the absolute differences between the SHR and HR estimates were ≤0.04; Table S17), confirming that the conclusions for CVD mortality were robust to the competing risk of non-cardiovascular death.

### Mediation analysis

**Figure 5.**
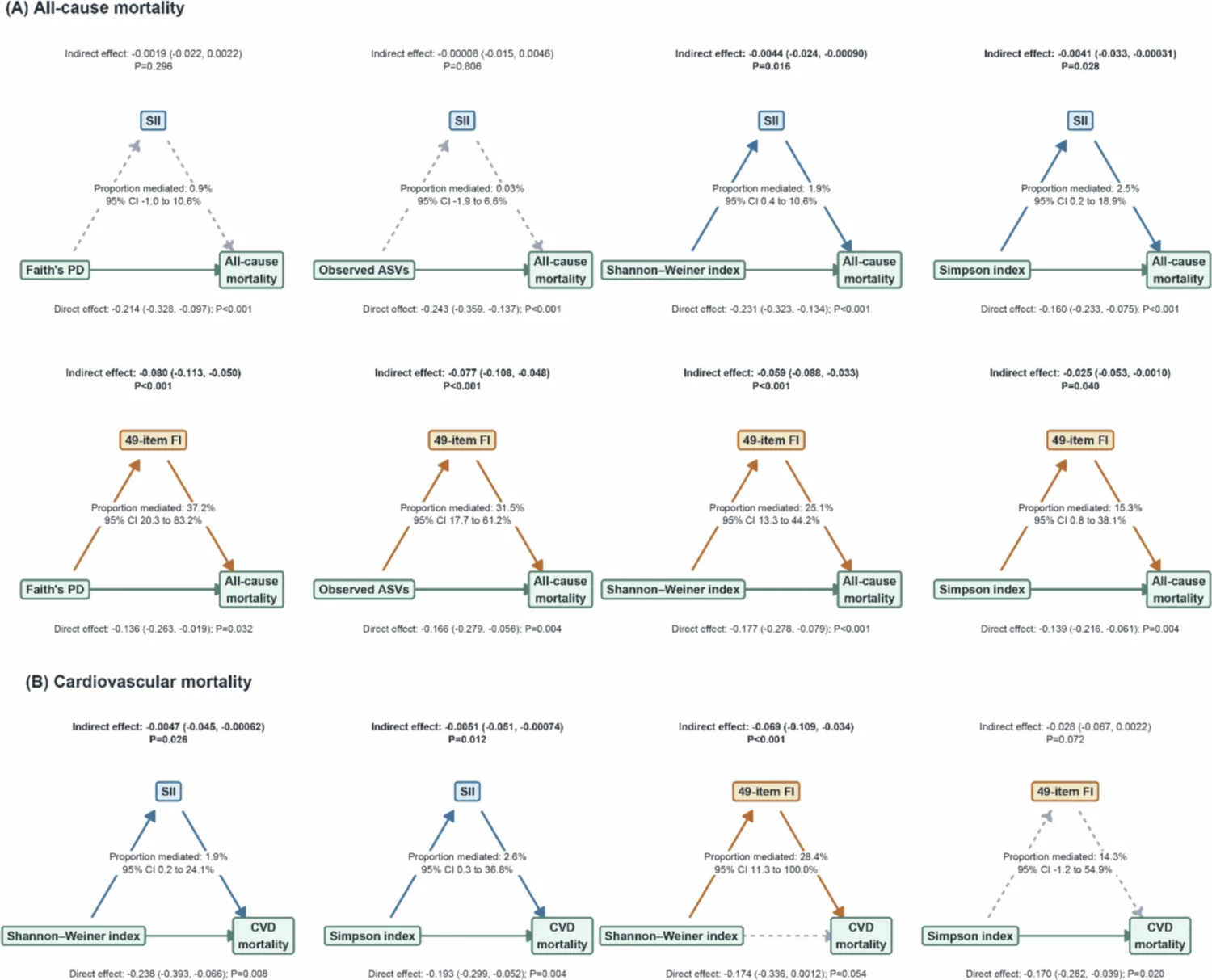
Mediation analysis of SII, frailty scores, and mortality in CKM patients. All-cause mortality (A), CVD mortality (B).

Exploratory mediation analyses yielded larger estimated indirect effects through the frailty index than through SII for the mortality associations examined. For all-cause mortality, the estimated proportions mediated through frailty ranged from 15.30% to 37.20% (Observed ASVs 31.50%, Faith's PD 37.20%, Shannon–Weiner index 25.10%, Simpson index 15.30%; all total *p* < 0.05, ACME *P* < 0.05). For CVD mortality, the estimated indirect proportions of frailty were 28.40% for Shannon–Weiner index effects (ACME *P* < 0.001), 14.3% for Simpson index (Figure 5). Indirect associations through SII were smaller and reached nominal statistical significance only for the Shannon–Weiner and Simpson indices.

## Discussion

The observed findings can be considered within a conceptual model in which oral microbial *α*-diversity, CKM burden, mortality, and frailty are interrelated. However, the present study does not establish the temporal or causal ordering of these factors. Lower *α*-diversity was associated cross-sectionally with advanced CKM and prospectively with all-cause and CVD mortality, while exploratory mediation analyses suggested that frailty-related measures may statistically account for part of mortality associations. Distinct facets of diversity tracked distinct points on this continuum—Faith's PD most strongly with advanced CKM, and evenness-based metrics (Shannon-Weiner and Simpson index) with CVD mortality. Our findings extend prior NHANES-based evidence linking oral microbial *α*-diversity to all-cause and CVD mortality [13] in three meaningful ways: by adopting the AHA CKM staging system as a primary outcome, by systematically comparing four complementary *α*-diversity metrics, and by exploring frailty as a potential mediator.

The differential patterns of *α*-diversity associations across CKM stage and mortality risk provide crucial biological insights. Faith’s PD and Observed ASVs demonstrated a linear association with advanced CKM, but an L-shaped, non-linear negative correlation with CKM-related all-cause mortality. This specific trajectory suggests that while the progressive loss of phylogenetic lineages tracks linearly with CKM severity, host survival is likely buffered by functional redundancy until richness drops below a critical threshold. Once this ecological tipping point is breached, mortality risk accelerates precipitously [31], underscoring that the extreme depletion of phylogenetically distant taxa marks a catastrophic broader community disruption. Conversely, Shannon–Weiner index, Simpson index exhibited consistent linear negative correlations with both all-cause and CVD mortality. This linear pattern indicates that the gradual loss of community equilibrium—potentially driven by the continuous expansion of dominant pathobionts at the expense of commensals [32]—proportionally increases cardiovascular risk by paradoxically homogenising community composition and compromising functional diversity. This metric-specificity has significant practical implications: studies examining only a single *α*-diversity index may fail to capture critical non-linear threshold effects or mischaracterise associations for which that metric is suboptimal sensitive [33]. Comprehensive characterisation across multiple complementary metrics is therefore warranted to fully decode the oral microbiome–CKM.

The consistency of *α*-diversity–mortality associations across pre-specified subgroups—including age, sex, race—supports the limited evidence of heterogeneity across the examined subgroups of these findings. Subgroup analyses identified a significant race/ethnicity-by-Observed ASVs interaction within the advanced CKM cohort, with Observed ASVs functioning as a particularly strong inverse correlate among non-Hispanic Black participants; notably, evenness-based metrics did not exhibit this differential effect. Beyond contributions from cultural dietary patterns and oral hygiene practices [34,35]. This specific dependence on absolute taxon richness may reflect distinct biological resilience mechanisms in the face of cumulative socio-environmental stressors and historical disparities in healthcare access—collectively encompassed by the social determinants of health (SDOH) framework [36]. In populations subject to higher allostatic load, preservation of a diverse microbial reservoir (i.e. high Observed ASVs) may provide the functional redundancy [37] required to buffer against progressive cardiometabolic dysregulation.

Exploratory mediation analyses yielded larger estimated indirect effects through the frailty index than through SII. This comparative pattern suggests that frailty-related processes may warrant further investigation. Several explanations may contribute to the numerical differences between the FI and SII. First, the FI integrates cognitive, functional, psychosocial, comorbidity, anthropometric, hospital utilisation, and laboratory domains—reflecting cumulative, multisystem physiological dysregulation, each component of which is potentially modulated by oral microbiome-derived metabolites and mucosal barrier integrity [38–40]. Second, oral microbial communities contribute to systemic nitric oxide bioavailability, dietary nutrient bioconversion, and immune-modulatory short-chain fatty acid production; these processes may be captured by a multidomain FI [41–43]. Third, frailty captures cumulative, low-grade dysregulation across multiple organ systems—a temporal and qualitative pattern that aligns more closely with the chronic, gradual nature of microbiome-mediated effects than do acute or peripheral inflammatory readouts [44,45]. Fourth, peripheral inflammation indices are derived from circulating blood cells and may not capture the locally acting mucosal and tissue-specific inflammatory pathways most relevant to oral microbiome-systemic disease relationships [5,46]. This finding aligns with recent NHANES-based evidence linking frailty status to CKM stage progression and mortality risk [47]. However, mediation analysis should be interpreted as an exploration. The 49-item frailty index includes multiple variables that overlap conceptually and clinically with mortality risk, so part of the estimated mediation may reflect conceptual overlap rather than a biologically distinct pathway. Second, oral microbial *α*-diversity and the FI were both ascertained at the same baseline examination; the temporal ordering required for causal mediation could not be established for the exposure–mediator link, and reciprocal or reverse relationships cannot be excluded.

This study has several methodological strengths. First, to our knowledge, this is the first study to adopt the AHA CKM staging system as a primary outcome in microbiome research, to compare four complementary *α*-diversity metrics across CKM endpoints systematically, and to perform an exploratory comparison of indirect-association estimates obtained using FI and SII as candidate statistical mediators. Second, the use of NHANES—a large, socio-demographically diverse sample drawn through a multistage probability design —encompasses wide demographic, socioeconomic, behavioural, and clinical variation, strengthening the breadth of the exposure–outcome associations examined. Third, the 16S rRNA sequencing was performed under a standardised protocol, with *α*-diversity metrics computed at a uniform rarefaction depth, ensuring comparability across participants. Fourth, the prespecified analytical framework integrated complementary parametric (logistic, Cox) and flexible non-parametric (RCS, GAM) approaches, allowing simultaneous quantification of effect magnitude and dose-response shape. Fifth, the mediation comparison of two conceptually distinct candidate statistical mediators implemented using a common analytical approach provided an exploratory quantitative comparison of the estimated indirect effects through frailty and SII. Finally, the robustness of findings across complete-case, cancer-exclusion, and lag-period sensitivity analyses provides triangulating evidence against major alternative explanations.

Several limitations should be acknowledged. First, this is an observational study, and it is difficult to determine the causal relationship between oral microbiota diversity and the risk of death. Prospective intervention studies are needed. Second, analyses were not survey-weighted. Although all design-related variables were included as covariates, residual departures from the full NHANES target population cannot be excluded, and population-level generalisation should be made with caution. Third, oral microbiome composition was characterised by 16S rRNA sequencing, which provides genus-level resolution and limited functional inference; species- or strain-level characterisation, as well as metagenomic profiling of microbial functional capacity, will be required to identify specific taxa or pathways driving the observed associations. Fourth, oral microbial composition was assessed at a single time point, which may not adequately capture longer-term exposure given the documented intra-individual temporal variability of the oral microbiome. Fifth, despite multivariable adjustment for a comprehensive covariate set and the use of multiple sensitivity analyses, residual confounding from unmeasured factors (e.g. medication use, oral hygiene behaviours, other socioeconomic determinants) cannot be excluded. Finally, generalisability of findings beyond the US adult population—particularly to populations with substantially different dietary, microbial, and healthcare contexts—requires confirmation in independent cohorts.

## Conclusion

In this observational cohort, elevated oral microbial *α*-diversity was inversely associated with advanced CKM and with all-cause and CVD mortality, with distinct *α*-diversity facets tracking distinct endpoints. Exploratory mediation analyses yielded larger estimated indirect effects through the frailty index than through SII. Oral microbial *α*-diversity may represent a risk-related correlate of cardiometabolic health, while frailty-related processes warrant further investigation in longitudinal and mechanistic studies. Future studies with repeated measurements and stronger temporal designs are needed to clarify directionality and causality.

## Supplementary Material

supplement.docx

## Data Availability

The data for this study can be accessed from the website at https://www.cdc.gov/nchs/nhanes/index.htm.
