# Supplementary material for: Unveiling the link between oral microbial α-diversity, advanced cardiovascular-kidney-metabolic syndrome, and mortality
Source: J Oral Microbiol. 2026 Aug 21;18(1):2717777. doi: 10.1080/20002297.2026.2717777 (PMC13501752; doi:10.1080/20002297.2026.2717777)
Supplement: Supplementary file 1 [file ZJOM_A_2717777_SM5616.docx]

Table S1. Definitions of CKM conditions.

| CKM conditions | Definition | CKM indicators | Threshold for CKM indicators |
| --- | --- | --- | --- |
| CVD | Individuals with clinical CVD or subclinical CVD | Clinical CVD | History of chronic heart failure, coronary heart disease, heart attack, or stroke |
|  |  | Subclinical CVD | Any of the following criteria is met:  (1) Very high-risk CKD in KDIGO classification: UACR ≥ 300 mg/g and eGFR ≤ 45-59 ml/min/1.73m^2^, UACR ≥ 30 mg/g and eGFR ≤ 30-44 ml/min/1.73m^2^, or eGFR ≤ 29 ml/min/1.73m^2^.  (2) Predicted 10-year CVD risk ≥ 20% |
| Kidney diseases | Individuals with CKD | CKD | Moderate-to-high-risk CKD in KDIGO classification: UACR ≥ 30 mg/g and eGFR ≥ 60 ml/min/1.73m^2^, UACR < 300 mg/g and eGFR ≤ 45-59 ml/min/1.73m^2^, or UACR < 30 mg/g and eGFR ≤ 30-44 ml/min/1.73m^2^. |
| Metabolic disorders | Individuals with overweight/obesity, abdominal obesity, prediabetes, diabetes, hypertension, hypertriglyceridemia or MetS | Overweight/obesity | BMI ≥25 kg/m^2^ (or ≥23 kg/m^2^ if Asian ancestry) |
|  |  | Abdominal obesity | Waist circumference ≥88/102 cm in female/male (or if Asian ancestry ≥80/90 cm in female/male) |
|  |  | Prediabetes | Fasting blood glucose ≥ 100-124 mg/dL or HbA1c ≥ 5.7%-6.4% and without self-reported diagnosis of diabetes, use of insulin, or oral hypoglycemic agents |
|  |  | Diabetes | Fasting blood glucose ≥ 125 mg/dL or HbA1c ≥ 6.5% or self-reported diagnosis of diabetes, use of insulin, or oral hypoglycemic agents |
|  |  | Hypertension | SBP ≥130 mm Hg or DBP ≥80 mm Hg or self-reported diagnosis of hypertension or use of antihypertensive medications |
|  |  | Hypertriglyceridemia | Triglycerides ≥ 135 mg/dL |
|  |  | MetS | MetS is defined by the presence of 3 or more of the following: (1) Waist circumference ≥88/102 cm in female/male (or if Asian ancestry ≥80/90 cm in female/male). (2) HDL cholesterol <50/40 mg/dL in females/males. (3) Triglycerides ≥150 mg/dL. (4) Elevated blood pressure (SBP ≥130 mm Hg or DBP ≥80 mm Hg and/or use of antihypertensive medications). (5) Fasting blood glucose ≥100 mg/dL. |

*Asian was not listed as a separate race/ethnicity until NAHNES 2011-2012; the uniform threshold for BMI and waist circumference was used in all participants in NHANES 1999-2010.

Table S2: Detailed algorithm for evaluating each CKM stage.

| CKM stages | Definition | Criterion | Threshold for CKM conditions |
| --- | --- | --- | --- |
| Stage 0: No CKM risk factors | Individuals with normal BMI and waist circumference, normoglycemia, normotension, a normal lipid profile, and no evidence of CKD or subclinical or clinical CVD | All criteria are met | BMI <25 kg/m^2^ (or <23 kg/m^2^ if Asian ancestry) |
|  |  |  | Waist circumference <88/102 cm in female/male (or if Asian ancestry <80/90 cm in female/male) |
|  |  |  | Fasting blood glucose < 100 mg/dL and HbA1c < 5.7%, and without self-reported diagnosis of diabetes, use of insulin, or oral hypoglycemic agents |
|  |  |  | SBP <130 mm Hg and DBP <80 mm Hg without self-reported diagnosis of hypertension or use of antihypertensive medications |
|  |  |  | HDL cholesterol >50/40 mg/dL in female/male and triglycerides < 150 mg/dL |
|  |  |  | Low-risk CKD in KDIGO classification according to eGFR and UACR: UACR < 30 mg/g and eGFR ≥ 60 ml/min/1.73m^2^. |
|  |  |  | Predicted 10-year CVD risk < 20% |
|  |  |  | No clinical CVD |
| Stage 1: Excess or dysfunctional adiposity | Individuals with overweight/obesity, abdominal obesity, or dysfunctional adipose tissue, without the presence of other metabolic risk factors or CKD | Any of the three criteria is met | Overweight/obesity |
|  |  |  | Abdominal obesity |
|  |  |  | Prediabetes |
|  |  | All criteria are met | SBP <130 mm Hg and DBP <80 mm Hg without self-reported diagnosis of hypertension or use of antihypertensive medications |
|  |  |  | HDL cholesterol >50/40 mg/dL in female/male and triglycerides <150 mg/dL |
|  |  |  | Low-risk CKD in KDIGO classification according to eGFR and UACR: UACR < 30 mg/g and eGFR ≥ 60 ml/min/1.73m^2^ |
|  |  |  | Predicted 10-year CVD risk < 20% |
|  |  |  | No clinical CVD |
| Stage 2: Metabolic risk factors and CKD | Individuals with metabolic risk factors (hypertriglyceridemia, hypertension, MetS, diabetes), or CKD | Any of the five criteria is met | Hypertriglyceridemia |
|  |  |  | Hypertension |
|  |  |  | diabetes |
|  |  |  | MetS |
|  |  |  | Moderate-to-high-risk CKD in KDIGO classification |
|  |  | All criteria are met | No very high-risk CKD in KDIGO classification |
|  |  |  | Predicted 10-year CVD risk < 20% |
|  |  |  | No clinical CVD |
| Stage 3: Subclinical CVD in CKM | Subclinical CVD among individuals with excess/dysfunctional adiposity, other metabolic risk factors, or CKD | Any of the two criteria is met | Very high-risk CKD in KDIGO classification |
|  |  |  | Predicted 10-year CVD risk ≥ 20% |
|  |  | Any of the eight criteria is met | Overweight/obesity |
|  |  |  | Abdominal obesity |
|  |  |  | Prediabetes |
|  |  |  | Hypertriglyceridemia |
|  |  |  | Hypertension |
|  |  |  | diabetes |
|  |  |  | MetS |
|  |  |  | Moderate-to-high-risk CKD in KDIGO classification |
|  |  | The criterion is met | No clinical CVD |
| Stage 4: Clinical CVD in CKM | Clinical CVD among individuals with excess/dysfunctional adiposity, other metabolic risk factors, or CKD | The criterion is met | Clinical CVD |
|  |  | Any of the nine criteria is met | Overweight/obesity |
|  |  |  | Abdominal obesity |
|  |  |  | Prediabetes |
|  |  |  | Hypertriglyceridemia |
|  |  |  | Hypertension |
|  |  |  | diabetes |
|  |  |  | MetS |
|  |  |  | Moderate-to-high-risk CKD in KDIGO classification |
|  |  |  | Very high-risk CKD in KDIGO classification |

Asian was not listed as a separate race/ethnicity until NHANES 2011-2012, the uniform threshold for BMI and waist circumference was used in all participants in NHANES 1999-2010.

Table S3: Components of the 49-item frailty index.

| Variable | Scoring | Variable in NHANES |
| --- | --- | --- |
| Cognition |  |  |
| 1. Experience confusion/memory problems | Yes = 1; No = 0 | PFQ057 |
| Dependence |  |  |
| 2. Managing money | No difficulty = 0; Some difficulty = 0.33; Much difficulty = 0.66; Unable to do = 1 | PFQ061a |
| 3. Stooping, crouching, kneeling difficulty | No difficulty = 0; Some difficulty = 0.33; Much difficulty = 0.66; Unable to do = 1 | PFQ061d |
| 4. Lifting or carrying difficulty | No difficulty = 0; Some difficulty = 0.33; Much difficulty = 0.66; Unable to do = 1 | PFQ061e |
| 5. House chore difficulty | No difficulty = 0; Some difficulty = 0.33; Much difficulty = 0.66; Unable to do = 1 | PFQ061f |
| 6. Preparing meals difficulty | No difficulty = 0; Some difficulty = 0.33; Much difficulty = 0.66; Unable to do = 1 | PFQ061g |
| 7. Standing up from armless chair difficulty | No difficulty = 0; Some difficulty = 0.33; Much difficulty = 0.66; Unable to do = 1 | PFQ061i |
| 8. Getting in and out of bed difficulty | No difficulty = 0; Some difficulty = 0.33; Much difficulty = 0.66; Unable to do = 1 | PFQ061j |
| 9. Using fork, knife, drinking from cup difficulty | No difficulty = 0; Some difficulty = 0.33; Much difficulty = 0.66; Unable to do = 1 | PFQ061k |
| 10. Dressing yourself difficulty | No difficulty = 0; Some difficulty = 0.33; Much difficulty = 0.66; Unable to do = 1 | PFQ061L |
| 11. Standing for long periods difficulty | No difficulty = 0; Some difficulty = 0.33; Much difficulty = 0.66; Unable to do = 1 | PFQ061m |
| 12. Grasp/holding small objects difficulty | No difficulty = 0; Some difficulty = 0.33; Much difficulty = 0.66; Unable to do = 1 | PFQ061p |
| 13. Attending social event difficulty | No difficulty = 0; Some difficulty = 0.33; Much difficulty = 0.66; Unable to do = 1 | PFQ061r |
| 14. Push or pull large objects difficulty | No difficulty = 0; Some difficulty = 0.33; Much difficulty = 0.66; Unable to do = 1 | PFQ061t |
| 15. Walking for a quarter mile difficulty | No difficulty = 0; Some difficulty = 0.33; Much difficulty = 0.66; Unable to do = 1 | PFQ061b |
| 16. Walking up 10 steps difficulty | No difficulty = 0; Some difficulty = 0.33; Much difficulty = 0.66; Unable to do = 1 | PFQ061c |
| 17. Leisure activity at home difficulty | No difficulty = 0; Some difficulty = 0.33; Much difficulty = 0.66; Unable to do = 1 | PFQ061s |
| Depressive Symptoms |  |  |
| 18. Have little interest in doing things | Nearly every day = 1, More than half the days = 0.66, Several days = 0.33, Not at all = 0 | DPQ010 |
| 19. Feeling down, depressed, or hopeless | Nearly every day = 1, More than half the days = 0.66, Several days = 0.33, Not at all = 0 | DPQ020 |
| 20. Trouble sleeping or sleeping too much | Nearly every day = 1, More than half the days = 0.66, Several days = 0.33, Not at all = 0 | DPQ030 |
| 21. Feeling tired or having little energy | Nearly every day = 1, More than half the days = 0.66, Several days = 0.33, Not at all = 0 | DPQ040 |
| 22. Poor appetite or overeating | Nearly every day = 1, More than half the days = 0.66, Several days = 0.33, Not at all = 0 | DPQ050 |
| 23. Feeling bad about yourself | Nearly every day = 1, More than half the days = 0.66, Several days = 0.33, Not at all = 0 | CIQD029  DPQ060 |
| 24. Trouble concentrating on things | Nearly every day = 1, More than half the days = 0.66, Several days = 0.33, Not at all = 0 | DPQ070 |
| Comorbidities |  |  |
| 25. Arthritis | Yes = 1, No = 0 | MCQ160A |
| 26. Thyroid problems | Yes = 1, No = 0 | MCQ160M |
| 27. Chronic bronchitis | Yes = 1, No = 0 | MCQ160K |
| 28. Cancer | Yes = 1, No = 0 | MCQ220 |
| 29. Congestive heart failure | Yes = 1, No = 0 | MCQ160B |
| 30. Coronary heart disease | Yes = 1, No = 0 | MCQ160C |
| 31. Angina/Angina pectoris | Yes = 1, No = 0 | MCQ160D |
| 32. Heart attack | Yes = 1, No = 0 | MCQ160E |
| 33. Stroke | Yes = 1, No = 0 | MCQ160F |
| 34. Hypertension | Yes = 1, No = 0 | BPQ020 |
| 35. Diabetes | Yes = 1, No = 0 | DIQ010 |
| 36. Weak/failing kidneys | Yes = 1, No = 0 | KIQ020  KIQ022 |
| 37. Urinary Leakage | Greatly = 1, Very much = 0.75, Somewhat = 0.5, Only a little = 0.25 | KIQ040  KIQ050 |
| Hospital Utilization and Access to Care |  |  |
| 38. Self-rated health | Fair, poor = 1, Excellent, Very good, good = 0 | HUQ010 |
| 39. Health now compared with 1 year ago | Worse = 1, About the same, better = 0 | HUQ020 |
| 40. Overnight hospital patient in past year | Yes = 1, No = 0 | HUQ070  HUD070  HUQ071 |
| 41. Frequency of health care use during past year | None = 0, 1-4 = 0.5, 5 and more = 1 | HUQ050  HUQ051 |
| 42. Number of prescribed medications | None = 0, 1-4 = 0.5, 5 and more = 1 | RXDCOUNT |
| Physical Anthropometry |  |  |
| 43. Body mass index | < 18.5, ≥30 = 1; 25-30 = 0.5; 18.5-25 = 0 | BMXBMI |
| Laboratory Values |  |  |
| 44. Glycohemoglobin (%) | 0%-5.7% = 0, >5.7% = 1 | LBXGH |
| 45. Red blood cell count (million cells/mL) | MALE: 4.7-6.1 = 0, Other = 1 FEMALE: 4.2-5.4 = 0, Other = 1 | LBXRBCSI |
| 46. Hemoglobin (g/dL) | MALE: 13.5-18 = 0, Other = 1 FEMALE: 12-16 = 0, Other = 1 | LBXHGB |
| 47. Red cell distribution width (%) | 11.6-14.6 = 0, Other = 1 | LBXRDW |
| 48. Lymphocyte percent (%) | 20-40 = 0, Other = 1 | LBXLYPCT |
| 49. Segmented neutrophils percent (%) | 40-80 = 0, Other = 1 | LBXNEPCT |

For each participant, the frailty index was computed as the proportion of deficits present among items with non-missing data; participants with fewer than 30 assessable items were excluded from frailty-related analyses.

Table S4: Proportions of missing values.

| Variable | N total | N missing | Missing (%) |
| --- | --- | --- | --- |
| Periodontitis | 6044 | 757 | 12.52 |
| Alcohol | 6044 | 512 | 8.47 |
| PIR | 6044 | 497 | 8.22 |
| Education | 6044 | 7 | 0.12 |
| Marital | 6044 | 4 | 0.07 |
| Smoking | 6044 | 2 | 0.03 |
| Age | 6044 | 0 | 0 |
| Sex | 6044 | 0 | 0 |
| Eth | 6044 | 0 | 0 |

Table S5: The cut points of α-Diversity metrics

| Sample | N | Metric | Min | Max | Q1 range | Q2 range | Q3 range | Q4 range |
| --- | --- | --- | --- | --- | --- | --- | --- | --- |
| Advanced CKM | 6044.00 | Observed ASVs | 10.20 | 352.90 | [10.2, 98.27] | (98.27, 125.8] | (125.8, 157.3] | (157.3, 352.9] |
| Advanced CKM | 6044.00 | Faith's PD | 2.52 | 85.52 | [2.52, 12.25] | (12.25, 14.44] | (14.44, 17.01] | (17.01, 85.51] |
| Advanced CKM | 6044.00 | Shannon–Weiner index | 0.82 | 6.60 | [0.82, 4.2] | (4.2, 4.65] | (4.65, 5.06] | (5.06, 6.6] |
| Advanced CKM | 6044.00 | Simpson index | 0.18 | 0.98 | [0.18, 0.88] | (0.88, 0.92] | (0.92, 0.94] | (0.94, 0.98] |
| All-cause Mortality | 6034.00 | Observed ASVs | 10.20 | 352.90 | [10.2, 98.3] | (98.3, 125.8] | (125.8, 157.28] | (157.28, 352.9] |
| All-cause Mortality | 6034.00 | Faith's PD | 2.52 | 85.52 | [2.52, 12.26] | (12.26, 14.43] | (14.43, 17] | (17, 85.51] |
| All-cause Mortality | 6034.00 | Shannon–Weiner index | 0.82 | 6.60 | [0.82, 4.2] | (4.2, 4.65] | (4.65, 5.06] | (5.06, 6.6] |
| All-cause Mortality | 6034.00 | Simpson index | 0.18 | 0.98 | [0.18, 0.88] | (0.88, 0.92] | (0.92, 0.94] | (0.94, 0.98] |
| CVD Mortality | 6034.00 | Observed ASVs | 10.20 | 352.90 | [10.2, 98.3] | (98.3, 125.8] | (125.8, 157.28] | (157.28, 352.9] |
| CVD Mortality | 6034.00 | Faith's PD | 2.52 | 85.52 | [2.52, 12.26] | (12.26, 14.43] | (14.43, 17] | (17, 85.51] |
| CVD Mortality | 6034.00 | Shannon–Weiner index | 0.82 | 6.60 | [0.82, 4.2] | (4.2, 4.65] | (4.65, 5.06] | (5.06, 6.6] |
| CVD Mortality | 6034.00 | Simpson index | 0.18 | 0.98 | [0.18, 0.88] | (0.88, 0.92] | (0.92, 0.94] | (0.94, 0.98] |

Table S6: The proportional hazards assumption.

| Outcome | Exposure | Exposure PH P | Global PH P | Exposure satisfies | Covariates violating |
| --- | --- | --- | --- | --- | --- |
| All-cause mortality | Shannon–Weiner index | 0.14 | 0.56 | Yes | None |
| CVD mortality | Shannon–Weiner index | 0.24 | 0.65 | Yes | None |
| All-cause mortality | Observed ASVs | 0.47 | 0.62 | Yes | None |
| CVD mortality | Observed ASVs | 0.54 | 0.74 | Yes | None |
| All-cause mortality | Faith's PD | 0.55 | 0.62 | Yes | None |
| CVD mortality | Faith's PD | 0.49 | 0.73 | Yes | None |
| All-cause mortality | Simpson index | 0.39 | 0.58 | Yes | None |
| CVD mortality | Simpson index | 0.33 | 0.66 | Yes | None |

Table S7: Variance inflation factor multicollinearity values for variables.

| variable | GVIF | Df | GVIF^(1/(2*Df)) |
| --- | --- | --- | --- |
| Shannon–Weiner index | 10.88 | 1 | 3.30 |
| Out richness | 8.86 | 1 | 2.97 |
| Faiths’PD | 5.90 | 1 | 2.40 |
| Simpson index | 5.75 | 1 | 2.40 |
| Age | 1.22 | 1 | 1.10 |
| Sex | 1.19 | 1 | 1.09 |
| Eth | 1.36 | 3 | 1.05 |
| Education | 1.40 | 2 | 1.09 |
| Smoking | 1.23 | 1 | 1.11 |
| Alcohol | 1.20 | 1 | 1.10 |
| Marital | 1.18 | 2 | 1.04 |
| PIR | 1.41 | 1 | 1.19 |
| Periodontitis | 1.36 | 1 | 1.17 |

Table S8: Knot selection of RCS analysis.

| Outcome | Metric | Knots | AIC | BIC | AIC best | BIC best | Final selected number of knots |
| --- | --- | --- | --- | --- | --- | --- | --- |
| Advanced CKM | Observed ASVs | 3 | 6473.46 | 6580.77 | * | * | 3 |
|  | Observed ASVs | 4 | 6474.93 | 6588.95 |  |  |  |
|  | Observed ASVs | 5 | 6474.48 | 6595.21 |  |  |  |
|  | Observed ASVs | 6 | 6475.51 | 6602.94 |  |  |  |
|  | Faith's PD | 3 | 6473.24 | 6580.54 |  | * | 3 |
|  | Faith's PD | 4 | 6472.83 | 6586.85 |  |  |  |
|  | Faith's PD | 5 | 6474.06 | 6594.79 |  |  |  |
|  | Faith's PD | 6 | 6472.33 | 6599.76 | * |  |  |
|  | Shannon–Weiner index | 3 | 6479.93 | 6587.24 | * | * | 3 |
|  | Shannon–Weiner index | 4 | 6480.94 | 6594.95 |  |  |  |
|  | Shannon–Weiner index | 5 | 6480.43 | 6601.15 |  |  |  |
|  | Shannon–Weiner index | 6 | 6481.06 | 6608.49 |  |  |  |
|  | Simpson index | 3 | 6478.15 | 6585.46 |  | * | 3 |
|  | Simpson index | 4 | 6479.94 | 6593.96 |  |  |  |
|  | Simpson index | 5 | 6480.77 | 6601.49 |  |  |  |
|  | Simpson index | 6 | 6477.92 | 6605.35 | * |  |  |
| All-cause Mortality | Observed ASVs | 3 | 6468 | 6568.58 | * | * | 3 |
|  | Observed ASVs | 4 | 6469.19 | 6576.47 |  |  |  |
|  | Observed ASVs | 5 | 6471.01 | 6585 |  |  |  |
|  | Observed ASVs | 6 | 6471.38 | 6592.07 |  |  |  |
|  | Faith's PD | 3 | 6473.58 | 6574.16 | * | * | 3 |
|  | Faith's PD | 4 | 6474.34 | 6581.62 |  |  |  |
|  | Faith's PD | 5 | 6476.27 | 6590.26 |  |  |  |
|  | Faith's PD | 6 | 6478.27 | 6598.96 |  |  |  |
|  | Shannon–Weiner index | 3 | 6469.47 | 6570.05 | * | * | 3 |
|  | Shannon–Weiner index | 4 | 6471.2 | 6578.49 |  |  |  |
|  | Shannon–Weiner index | 5 | 6473.23 | 6587.22 |  |  |  |
|  | Shannon–Weiner index | 6 | 6474.53 | 6595.23 |  |  |  |
|  | Simpson index | 3 | 6476.49 | 6577.07 | * | * | 3 |
|  | Simpson index | 4 | 6478.3 | 6585.58 |  |  |  |
|  | Simpson index | 5 | 6479.73 | 6593.72 |  |  |  |
|  | Simpson index | 6 | 6481.58 | 6602.27 |  |  |  |
| CVD Mortality | Observed ASVs | 3 | 2303.66 | 2404.24 | * | * | 3 |
|  | Observed ASVs | 4 | 2305.66 | 2412.95 |  |  |  |
|  | Observed ASVs | 5 | 2307.64 | 2421.63 |  |  |  |
|  | Observed ASVs | 6 | 2305.29 | 2425.99 |  |  |  |
|  | Faith's PD | 3 | 2305.6 | 2406.18 | * | * | 3 |
|  | Faith's PD | 4 | 2307.13 | 2414.41 |  |  |  |
|  | Faith's PD | 5 | 2309.15 | 2423.14 |  |  |  |
|  | Faith's PD | 6 | 2310.42 | 2431.11 |  |  |  |
|  | Shannon–Weiner index | 3 | 2300.9 | 2401.48 | * | * | 3 |
|  | Shannon–Weiner index | 4 | 2302.81 | 2410.09 |  |  |  |
|  | Shannon–Weiner index | 5 | 2304.58 | 2418.57 |  |  |  |
|  | Shannon–Weiner index | 6 | 2306.13 | 2426.82 |  |  |  |
|  | Simpson index | 3 | 2302.14 | 2402.72 | * | * | 3 |
|  | Simpson index | 4 | 2304.22 | 2411.5 |  |  |  |
|  | Simpson index | 5 | 2305.28 | 2419.27 |  |  |  |
|  | Simpson index | 6 | 2306.4 | 2427.09 |  |  |  |

Table S9: The all-cause and CVD death counts across diversity categories.

|  |  |  | All-cause | | CVD | |
| --- | --- | --- | --- | --- | --- | --- |
| Metric | Quartile | N_at_risk | Events_n | Incidence_per_1000PY | Events_n | Incidence_per_1000PY |
| Observed ASVs | Q1(Lowest) | 1508 | 154 | 11.99 | 52 | 4.05 |
| Observed ASVs | Q2 | 1510 | 88 | 6.66 | 34 | 2.57 |
| Observed ASVs | Q3 | 1508 | 77 | 5.78 | 25 | 1.88 |
| Observed ASVs | Q4(Highest) | 1508 | 83 | 6.2 | 33 | 2.46 |
| Faith's PD | Q1(Lowest) | 1508 | 153 | 11.87 | 53 | 4.11 |
| Faith's PD | Q2 | 1510 | 83 | 6.28 | 32 | 2.42 |
| Faith's PD | Q3 | 1509 | 81 | 6.1 | 27 | 2.03 |
| Faith's PD | Q4(Highest) | 1507 | 85 | 6.36 | 32 | 2.39 |
| Shannon–Weiner index | Q1(Lowest) | 1507 | 146 | 11.18 | 53 | 4.06 |
| Shannon–Weiner index | Q2 | 1509 | 93 | 7.09 | 35 | 2.67 |
| Shannon–Weiner index | Q3 | 1508 | 84 | 6.36 | 25 | 1.89 |
| Shannon–Weiner index | Q4(Highest) | 1510 | 79 | 5.91 | 31 | 2.32 |
| Simpson index | Q1(Lowest) | 1507 | 123 | 9.35 | 45 | 3.42 |
| Simpson index | Q2 | 1508 | 115 | 8.82 | 40 | 3.07 |
| Simpson index | Q3 | 1511 | 93 | 7.04 | 33 | 2.5 |
| Simpson index | Q4(Highest) | 1508 | 71 | 5.32 | 26 | 1.95 |

Table S10: Primary metric–outcome associations (continuous, per 1-SD, fully adjusted Model 3) with Benjamini–Hochberg FDR-adjusted q values.

| Metric | Outcome | OR/HR | 95%CI | P value | FDR Q all | FDR Q by family |
| --- | --- | --- | --- | --- | --- | --- |
| Observed ASVs | Advanced CKM | 0.92 | (0.86-0.98) | 0.009 | 0.01 | 0.02 |
| Faith's PD | Advanced CKM | 0.91 | (0.86-0.98) | 0.009 | 0.01 | 0.02 |
| Shannon–Weiner index | Advanced CKM | 0.99 | (0.93-1.05) | 0.76 | 0.76 | 0.76 |
| Simpson index | Advanced CKM | 1.05 | (0.99-1.12) | 0.13 | 0.15 | 0.18 |
| Observed ASVs | All-cause mortality | 0.78 | (0.70-0.87) | <0.001 | 0.002 | 0.001 |
| Faith's PD | All-cause mortality | 0.80 | (0.72-0.90) | <0.001 | 0.002 | 0.001 |
| Shannon–Weiner index | All-cause mortality | 0.79 | (0.72-0.86) | <0.001 | 0.002 | 0.001 |
| Simpson index | All-cause mortality | 0.85 | (0.78-0.92) | <0.001 | 0.002 | 0.001 |
| Observed ASVs | CVD mortality | 0.81 | (0.68-0.96) | 0.02 | 0.02 | 0.02 |
| Faith's PD | CVD mortality | 0.86 | (0.72-1.03) | 0.10 | 0.11 | 0.09 |
| Shannon–Weiner index | CVD mortality | 0.79 | (0.68-0.92) | 0.003 | 0.01 | 0.01 |
| Simpson index | CVD mortality | 0.82 | (0.72-0.93) | 0.003 | 0.01 | 0.01 |

Table S11: Subgroup analysis for advanced CKM.

|  | Observed ASVs | |  |  |  | Faith's PD | |
| --- | --- | --- | --- | --- | --- | --- | --- |
| Subgroup | OR (95%CI) | | P-value | P -interaction | OR (95%CI) | P-value | P-interaction |
| Male | 0.91(0.83-0.99) | | 0.03 | 0.14 | 0.89(0.81-0.97) | 0.01 | 0.06 |
| Female | 1.00(0.91-1.08) | | 1.00 |  | 1.00(0.92-1.09) | 0.99 |  |
| Age<65 | 0.94(0.88-1.01) | | 0.09 | 0.65 | 0.94(0.88-1.00) | 0.06 | 0.57 |
| Age≥65 | 0.97(0.81-1.15) | | 0.69 |  | 0.98(0.82-1.16) | 0.78 |  |
| Non-Hispanic White | 0.95(0.85-1.06) | | 0.33 | 0.04 | 0.93(0.84-1.03) | 0.18 | 0.54 |
| Non-Hispanic Black | 0.85(0.75-0.95) | | 0.01 |  | 0.90(0.81-1.01) | 0.08 |  |
| Mexican American | 1.06(0.92-1.23) | | 0.43 |  | 1.03(0.88-1.21) | 0.72 |  |
| Hispanic and Other | 1.06(0.91-1.23) | | 0.48 |  | 1.01(0.87-1.18) | 0.88 |  |
| Male |  |  |  |  |  |  |  |
| Q1 | Ref |  |  |  |  |  |  |
| Q2 | 0.90(0.71-1.15) | | 0.41 |  | 0.74(0.58-0.94) | 0.01 |  |
| Q3 | 0.82(0.64-1.04) | | 0.10 |  | 0.812(0.64-1.04) | 0.10 |  |
| Q4 | 0.81(0.64-1.03) | | 0.09 | 0.36 | 0.68(0.53-0.87) | 0.00 | 0.21 |
| Female |  |  |  |  |  |  |  |
| Q1 | Ref |  |  |  |  |  |  |
| Q2 | 0.89(0.71-1.11) | | 0.30 |  | 0.99(0.79-1.24) | 0.91 |  |
| Q3 | 0.931(0.737-1.18) | | 0.55 |  | 0.92(0.72-1.16) | 0.47 |  |
| Q4 | 1.051(0.82-1.35) | | 0.70 |  | 0.94(0.73-1.21) | 0.62 |  |
| Age<65 |  |  |  |  |  |  |  |
| Q1 | Ref |  |  |  |  |  |  |
| Q2 | 0.90(0.75-1.08) | | 0.25 |  | 0.90(0.75-1.08) | 0.25 |  |
| Q3 | 0.87(0.73-1.04) | | 0.13 |  | 0.85(0.71-1.02) | 0.07 |  |
| Q4 | 0.90(0.75-1.09) | | 0.28 | 0.93 | 0.78(0.65-0.94) | 0.01 | 0.07 |
| Age≥65 |  |  |  |  |  |  |  |
| Q1 | Ref |  |  |  |  |  |  |
| Q2 | 0.88(0.58-1.33) | | 0.53 |  | 0.64(0.42-0.97) | 0.03 |  |
| Q3 | 0.89(0.57-1.39) | | 0.60 |  | 1.13(0.72-1.78) | 0.58 |  |
| Q4 | 1.01(0.61-1.67) | | 0.96 |  | 0.94(0.57-1.55) | 0.80 |  |
| Non-Hispanic White | | |  |  |  |  |  |
| Q1 | Ref |  |  |  |  |  |  |
| Q2 | 0.86(0.67-1.10) | | 0.22 |  | 0.74(0.58-0.94) | 0.02 |  |
| Q3 | 0.82(0.63-1.07) | | 0.15 |  | 0.79(0.6-1.03) | 0.08 |  |
| Q4 | 0.98(0.73-1.31) | | 0.89 | 0.09 | 0.83(0.62-1.11) | 0.21 | 0.50 |
| Non-Hispanic Black | |  |  |  |  |  |  |
| Q1 | Ref |  |  |  |  |  |  |
| Q2 | 0.86(0.62-1.19) | | 0.36 |  | 0.98(0.70-1.36) | 0.89 |  |
| Q3 | 0.90(0.65-1.25) | | 0.52 |  | 0.79(0.56-1.10) | 0.16 |  |
| Q4 | 0.64(0.46-0.89) | | 0.01 |  | 0.70(0.50-0.98) | 0.04 |  |
| Mexican American | |  |  |  |  |  |  |
| Q1 | Ref |  |  |  |  |  |  |
| Q2 | 0.85(0.52-1.39) | | 0.52 |  | 1.00(0.62-1.61) | 0.99 |  |
| Q3 | 0.95(0.60-1.50) | | 0.81 |  | 1.17(0.75-1.83) | 0.50 |  |
| Q4 | 1.01(0.64-1.60) | | 0.95 |  | 0.95(0.61-1.50) | 0.83 |  |
| Hispanic and Other | |  |  |  |  |  |  |
| Q1 | Ref |  |  |  |  |  |  |
| Q2 | 1.1(0.75-1.61) | | 0.62 |  | 0.96(0.66-1.41) | 0.84 |  |
| Q3 | 0.87(0.58-1.29) | | 0.48 |  | 0.98(0.67-1.43) | 0.92 |  |
| Q4 | 1.35(0.91-1.99) | | 0.14 |  | 0.93(0.62-1.38) | 0.71 |  |

Table S12: subgroup analysis for all-cause mortality.

|  | Observed ASVs | |  | Faith's PD | |  | Shannon–Weiner index | |  | Simpson index | |  |
| --- | --- | --- | --- | --- | --- | --- | --- | --- | --- | --- | --- | --- |
| Subgroup | HR (95%) | P value | P interaction | HR (95%) | P value | P interaction | HR (95%) | P value | P interaction | HR (95%) | P value | P interaction |
| Male | 0.77(0.67-0.88) | <0.001 | 0.66 | 0.81(0.70-0.92) | 0.002 | 0.71 | 0.8(0.71-0.90) | <0.001 | 0.82 | 0.88(0.80-0.97) | 0.01 | 0.66 |
| Female | 0.82(0.69-0.97) | 0.02 |  | 0.83(0.69-0.99) | 0.047 |  | 0.78(0.68-0.91) | 0.001 |  | 0.83(0.73-0.94) | 0.003 |  |
| Age<65 | 0.80(0.71-0.90) | <0.001 | 0.88 | 0.83(0.74-0.94) | 0.004 | 0.54 | 0.79(0.71-0.88) | <0.001 | 0.83 | 0.85(0.78-0.93) | <0.001 | 0.88 |
| Age≥65 | 0.73(0.59-0.91) | 0.005 |  | 0.72(0.58-0.90) | 0.003 |  | 0.76(0.63-0.93) | 0.001 |  | 0.83(0.70-0.99) | 0.04 |  |
| Non-Hispanic White | 0.75(0.64-0.87) | <0.001 | 0.12 | 0.81(0.687-0.955) | 0.01 | 0.17 | 0.74(0.65-0.85) | <0.001 | 0.17 | 0.82(0.74-0.90) | <0.001 | 0.27 |
| Non-Hispanic Black | 0.90(0.74-1.09) | 0.28 |  | 0.87(0.71-1.08) | 0.20 |  | 0.91(0.76-1.10) | 0.33 |  | 0.97(0.80-1.18) | 0.79 |  |
| Mexican American | 0.63(0.47-0.86) | 0.003 |  | 0.70(0.53-0.95) | 0.02 |  | 0.79(0.61-1.03) | 0.08 |  | 0.95(0.73-1.25) | 0.73 |  |
| Hispanic and Other | 0.77(0.59-1.00) | 0.05 |  | 0.72(0.55-0.94) | 0.02 |  | 0.72(0.56-0.91) | 0.006 |  | 0.75(0.61-0.93) | 0.01 |  |
| Male |  |  |  |  |  |  |  |  |  |  |  |  |
| Q1 |  |  |  |  |  |  |  |  |  |  |  |  |
| Q2 | 0.76(0.55-1.07) | 0.11 |  | 0.67(0.48-0.94) | 0.02 |  | 0.83(0.59-1.16) | 0.28 |  | 0.94(0.67-1.31) | 0.71 |  |
| Q3 | 0.612(0.43-0.871) | 0.006 |  | 0.69(0.49-0.97) | 0.03 |  | 0.76(0.54-1.06) | 0.10 |  | 0.87(0.62-1.23) | 0.42 |  |
| Q4 | 0.56(0.38-0.81) | 0.002 |  | 0.55(0.38-0.79) | 0.001 |  | 0.53(0.36-0.76) | <0.001 |  | 0.62(0.43-0.90) | 0.01 |  |
| Female |  |  | 0.28 |  |  | 0.11 |  |  | 0.21 |  |  | 0.67 |
| Q1 |  |  |  |  |  |  | 0.70(0.46-1.06) | 0.09 |  |  |  |  |
| Q2 | 0.66(0.43-1.02) | 0.03 |  | 0.65(0.42-1.00) | 0.05 |  | 0.55(0.34-0.89) | 0.02 |  | 0.94(0.63-1.41) | 0.78 |  |
| Q3 | 0.54(0.34-0.87) | 0.01 |  | 0.53(0.33-0.88) | 0.01 |  | 0.73(0.46-1.16) | 0.18 |  | 0.67(0.427-1.05) | 0.08 |  |
| Q4 | 0.81(0.5-1.31) | 0.39 |  | 0.90(0.57-1.44) | 0.67 |  |  |  |  | 0.48(0.29-0.82) | 0.01 |  |
| Age<65 |  |  |  |  |  |  |  |  |  |  |  |  |
| Q1 |  |  |  |  |  |  |  |  |  |  |  |  |
| Q2 | 0.75(0.55-1.02) | 0.07 |  | 0.66(0.48-0.90) | 0.01 |  | 0.81(0.60-1.09) | 0.17 |  | 0.85(0.63-1.15) | 0.30 |  |
| Q3 | 0.62(0.45-0.85) | 0.003 |  | 0.63(0.45-0.86) | 0.004 |  | 0.71(0.52-0.98) | 0.03 |  | 0.84(0.61-1.14) | 0.25 |  |
| Q4 | 0.59(0.42-0.83) | 0.002 |  | 0.64(0.46-0.88) | 0.007 |  | 0.60(0.43-0.84) | 0.003 |  | 0.55(0.39-0.78) | 0.01 |  |
| Age≥65 |  |  | 0.59 |  |  | 0.97 |  |  | 0.93 |  |  | 0.31 |
| Q1 |  |  |  |  |  |  |  |  |  |  |  |  |
| Q2 | 0.67(0.40-1.14) | 0.14 |  | 0.63(0.37-1.06) | 0.08 |  | 0.64(0.37-1.10) | 0.11 |  | 1.18(0.71-1.96) | 0.52 |  |
| Q3 | 0.49(0.26-0.90) | 0.02 |  | 0.68(0.39-1.18) | 0.17 |  | 0.56(0.31-1.00) | 0.05 |  | 0.62(0.34-1.13) | 0.12 |  |
| Q4 | 0.72(0.40-1.32) | 0.29 |  | 0.56(0.30-1.07) | 0.08 |  | 0.50(0.28-0.91) | 0.02 |  | 0.60(0.33-1.12) | 0.11 |  |
| Non-Hispanic White |  |  |  |  |  |  |  |  |  |  |  |  |
| Q1 |  |  |  |  |  |  |  |  |  |  |  |  |
| Q2 | 0.6(0.40-0.90) | 0.01 |  | 0.58(0.39-0.86) | 0.007 |  | 0.73(0.5-1.08) | 0.11 |  | 0.86(0.59-1.26) | 0.45 |  |
| Q3 | 0.49(0.32-0.75) | 0.001 |  | 0.64(0.42-0.96) | 0.03 |  | 0.61(0.40-0.91) | 0.01 |  | 0.65(0.43-0.97) | 0.04 |  |
| Q4 | 0.57(0.36-0.92) | 0.02 |  | 0.64(0.41-1.02) | 0.06 |  | 0.52(0.33-0.82) | 0.005 |  | 0.57(0.36-0.89) | 0.02 |  |
| Non-Hispanic Black |  |  | 0.47 |  |  | 0.87 |  |  | 0.63 |  |  | 0.74 |
| Q1 |  |  |  |  |  |  |  |  |  |  |  |  |
| Q2 | 1.14(0.69-1.89) | 0.60 |  | 0.80(0.47-1.34) | 0.39 |  | 0.96(0.58-1.58) | 0.86 |  | 1.19(0.73-1.93) | 0.49 |  |
| Q3 | 0.73(0.41-1.29) | 0.27 |  | 0.72(0.42-1.23) | 0.23 |  | 0.91(0.54-1.54) | 0.74 |  | 1.10(0.65-1.86) | 0.72 |  |
| Q4 | 0.86(0.50-1.47) | 0.57 |  | 0.74(0.44-1.24) | 0.25 |  | 0.71(0.42-1.20) | 0.20 |  | 0.68(0.40-1.17) | 0.17 |  |
| Mexican American |  |  |  |  |  |  |  |  |  |  |  |  |
| Q1 |  |  |  |  |  |  |  |  |  |  |  |  |
| Q2 | 0.46(0.21-1.05) | 0.06 |  | 0.49(0.21-1.15) | 0.10 |  | 0.84(0.35-2.00) | 0.69 |  | 1.22(0.51-2.94) | 0.65 |  |
| Q3 | 0.37(0.17-0.81) | 0.01 |  | 0.39(0.18-0.86) | 0.02 |  | 0.93(0.41-2.08) | 0.85 |  | 1.35(0.58-3.16) | 0.49 |  |
| Q4 | 0.34(0.16-0.76) | 0.008 |  | 0.47(0.22-1.02) | 0.06 |  | 0.55(0.23-1.30) | 0.18 |  | 0.7(0.26-1.70) | 0.40 |  |
| Hispanic and Other |  |  |  |  |  |  |  |  |  |  |  |  |
| Q1 |  |  |  |  |  |  |  |  |  |  |  |  |
| Q2 | 0.73(0.36-1.48) | 0.39 |  | 0.794(0.408-1.543) | 0.50 |  | 0.59(0.30-1.16) | 0.12 |  | 0.66(0.34-1.28) | 0.22 |  |
| Q3 | 0.87(0.43-1.75) | 0.69 |  | 0.63(0.30-1.33) | 0.23 |  | 0.33(0.14-0.77) | 0.01 |  | 0.48(0.23-1.00) | 0.05 |  |
| Q4 | 0.59(0.26-1.35) | 0.21 |  | 0.46(0.19-1.07) | 0.07 |  | 0.56(0.27-1.16) | 0.12 |  | 0.40(0.18-0.91) | 0.03 |  |

Table S13: Subgroup analysis for CVD mortality.

|  | Shannon–Weiner index |  |  | Simpson index |  |  |
| --- | --- | --- | --- | --- | --- | --- |
| Subgroup | HR (95%) | P value | P interaction | HR (95%) | P value | P interaction |
| Female | 0.88(0.68-1.14) | 0.33 | 0.28 | 0.87(0.69-1.10) | 0.25 | 0.43 |
| Male | 0.76(0.63-0.92) | 0.00 |  | 0.81(0.71-0.94) | 0.00 |  |
| Age<65 | 0.74(0.62-0.88) | <0.001 | 0.05 | 0.78(0.69-0.89) | <0.001 | 0.06 |
| Age≥65 | 0.97(0.69-1.36) | 0.86 |  | 1.07(0.74-1.56) | 0.71 |  |
| Non-Hispanic White | 0.76(0.60-0.95) | 0.02 | 0.58 | 0.80(0.66-0.95) | 0.01 | 0.88 |
| Non-Hispanic Black | 0.88(0.67-1.16) | 0.36 |  | 0.91(0.69-1.19) | 0.48 |  |
| Mexican American | 0.71(0.45-1.13) | 0.15 |  | 0.81(0.56-1.15) | 0.23 |  |
| Hispanic and Other | 0.76(0.51-1.13) | 0.18 |  | 0.78(0.55-1.12) | 0.17 |  |
| Female |  |  |  |  |  |  |
| Q1 |  |  |  |  |  |  |
| Q2 | 1.07(0.54-2.10) | 0.86 |  | 0.91(0.46-1.78) | 0.78 |  |
| Q3 | 0.60(0.26-1.41) | 0.24 |  | 0.73(0.35-1.52) | 0.40 |  |
| Q4 | 0.98(0.46-2.12) | 0.97 |  | 0.54(0.23-1.25) | 0.15 |  |
| Male |  |  | 0.44 |  |  | 0.98 |
| Q1 |  |  |  |  |  |  |
| Q2 | 0.71(0.40-1.26) | 0.24 |  | 0.89(0.51-1.54) | 0.67 |  |
| Q3 | 0.56(0.31-1.02) | 0.06 |  | 0.89(0.50-1.59) | 0.69 |  |
| Q4 | 0.50(0.27-0.90) | 0.02 |  | 0.60(0.32-1.10) | 0.10 |  |
| Age<65 |  |  |  |  |  |  |
| Q1 |  |  |  |  |  |  |
| Q2 | 0.77(0.47-1.26) | 0.30 |  | 0.75(0.45-1.20) | 0.22 |  |
| Q3 | 0.49(0.28-0.88) | 0.02 |  | 0.75(0.45-1.26) | 0.28 |  |
| Q4 | 0.56(0.32-0.97) | 0.04 |  | 0.43(0.23-0.78) | 0.01 |  |
| Age≥65 |  |  | 0.34 |  |  | 0.10 |
| Q1 |  |  |  |  |  |  |
| Q2 | 0.91(0.37-2.23) | 0.83 |  | 1.79(0.71-4.49) | 0.22 |  |
| Q3 | 0.84(0.33-2.11) | 0.71 |  | 1.08(0.40-2.93) | 0.89 |  |
| Q4 | 0.77(0.31-1.91) | 0.57 |  | 1.27(0.48-3.34) | 0.63 |  |
| Non-Hispanic White |  |  |  |  |  |  |
| Q1 |  |  |  |  |  |  |
| Q2 | 0.56(0.27-1.16) | 0.12 |  | 0.76(0.38-1.52) | 0.43 |  |
| Q3 | 0.48(0.22-1.04) | 0.06 |  | 0.60(0.28-1.28) | 0.18 |  |
| Q4 | 0.73(0.35-1.53) | 0.40 |  | 0.93(0.45-1.91) | 0.85 |  |
| Non-Hispanic Black |  |  | 0.81 |  |  | 0.51 |
| Q1 |  |  |  |  |  |  |
| Q2 | 1.32(0.64-2.71) | 0.45 |  | 1.21(0.59-2.50) | 0.61 |  |
| Q3 | 0.72(0.30-1.72) | 0.46 |  | 1.16(0.54-2.50) | 0.70 |  |
| Q4 | 0.75(0.34-1.66) | 0.48 |  | 0.54(0.23-1.26) | 0.15 |  |
| Mexican American |  |  |  |  |  |  |
| Q1 |  |  |  |  |  |  |
| Q2 | 0.48(0.10-2.29) | 0.36 |  | 1.47(0.35-6.19) | 0.60 |  |
| Q3 | 0.50(0.12-2.08) | 0.34 |  | 0.80(0.17-3.69) | 0.77 |  |
| Q4 | 0.33(0.08-1.42) | 0.14 |  | 0.30(0.05-1.91) | 0.20 |  |
| Hispanic and Other |  |  |  |  |  |  |
| Q1 |  |  |  |  |  |  |
| Q2 | 0.88(0.29-2.68) | 0.83 |  | 0.60(0.19-1.95) | 0.40 |  |
| Q3 | 0.64(0.18-2.26) | 0.49 |  | 0.71(0.23-2.17) | 0.55 |  |
| Q4 | 0.43(0.11-1.71) | 0.23 |  | 0.28(0.06-1.34) | 0.11 |  |

Table S14: Sensitive analysis of complete case analysis.

|  | Continuous OR/HR (95%CI), P | Q4 vs Q1 OR/HR (95%CI), P | P trend |
| --- | --- | --- | --- |
| Advanced CKM |  |  |  |
| Observed ASVs | 0.92 (0.85-0.99), P=0.03 | 0.77 (0.62-0.96), P=0.02 | 0.046 |
| Faith's PD | 0.89 (0.82-0.96), P=0.01 | 0.68 (0.54-0.85), P<0.001 | 0.003 |
| Shannon–Weiner index | 1.00 (0.93-1.08), P=0.98 | 0.94 (0.76-1.15), P=0.53 | 0.79 |
| Simpson index | 1.07 (0.99-1.15), P=0.10 | 1.18 (0.96-1.45), P=0.11 | 0.12 |
| All-cause mortality | |  |  |
| Observed ASVs | 0.78 (0.70-0.87), P<0.001 | 0.61 (0.45-0.82), P=0.001 | <0.001 |
| Faith's PD | 0.80 (0.72-0.90), P<0.001 | 0.63 (0.47-0.84), P=0.002 | <0.001 |
| Shannon–Weiner index | 0.79 (0.72-0.86), P<0.001 | 0.58 (0.43-0.77), P<0.001 | <0.001 |
| Simpson index | 0.845(0.78-0.92), P<0.001 | 0.57 (0.42-0.77), P<0.001 | <0.001 |
| CVD mortality |  |  |  |
| Observed ASVs | 0.82 (0.68-0.96), P=0.016 | 0.70 (0.43-1.14), P=0.155 | 0.062 |
| Faith's PD | 0.86 (0.72-1.03), P=0.10 | 0.657 (0.41-1.06), P=0.088 | 0.048 |
| Shannon–Weiner index | 0.79 (0.68-0.93), P=0.003 | 0.62 (0.39-0.99), P=0.048 | 0.018 |
| Simpson index | 0.82 (0.72-0.93), P=0.003 | 0.57 (0.35-0.93), P=0.02 | 0.023 |

Table S15: Sensitive analysis of excluding patients with cancer.

|  | Continuous OR/HR (95%CI), P | Q4 vs Q1 OR/HR (95%CI), P | P trend |
| --- | --- | --- | --- |
| Advanced CKM |  |  |  |
| Observed ASVs | 0.93 (0.86-0.99), P=0.03 | 0.85 (0.70-1.03), P=0.09 | 0.048 |
| Faith's PD | 0.93 (0.86-0.99), P=0.03 | 0.73 (0.61-0.89), P=0.001 | 0.002 |
| Shannon–Weiner index | 1.01 (0.94-1.08), P=0.86 | 1.00 (0.83-1.20), P=0.98 | 0.52 |
| Simpson index | 1.09 (1.01-1.16), P=0.02 | 1.20 (1.00-1.44), P=0.05 | 0.08 |
| All-cause mortality | |  |  |
| Observed ASVs | 0.78 (0.69-0.87), P<0.001 | 0.58 (0.42-0.80), P<0.001 | <0.001 |
| Faith's PD | 0.8 (0.71-0.90), P<0.001 | 0.62 (0.46-0.85), P=0.003 | <0.001 |
| Shannon–Weiner index | 0.80 (0.72-0.89), P<0.001 | 0.60 (0.44-0.82), P=0.001 | <0.001 |
| Simpson index | 0.86(0.79-0.95), P=0.001 | 0.6 (0.43-0.83), P=0.002 | 0.001 |
| CVD mortality |  |  |  |
| Observed ASVs | 0.8 (0.67-0.96), P=0.02 | 0.71 (0.43-1.16), P=0.17 | 0.08 |
| Faith's PD | 0.86 (0.71-1.03), P=0.10 | 0.65 (0.40-1.07), P=0.09 | 0.06 |
| Shannon–Weiner index | 0.79 (0.68-0.94), P=0.01 | 0.65 (0.40-1.06), P=0.09 | 0.03 |
| Simpson index | 0.83 (0.72-0.96), P=0.01 | 0.58 (0.35-0.97), P=0.04 | 0.03 |

Table S16: Sensitive analysis of excluding patients who died within the first two-year follow-up.

|  | Continuous OR/HR (95%CI), P | Q4 vs Q1 OR/HR (95%CI), P | P trend |
| --- | --- | --- | --- |
| Advanced CKM |  |  |  |
| Observed ASVs | 0.92 (0.86-0.98), P=0.01 | 0.82 (0.68-0.99), P=0.04 | 0.03 |
| Faith's PD | 0.92 (0.86-0.99), P=0.02 | 0.71 (0.59-0.86), P=<0.001 | <0.001 |
| Shannon–Weiner index | 1.00 (0.93-1.06), P=0.89 | 0.98(0.82-1.17), P=0.81 | 0.73 |
| Simpson index | 1.06 (0.99-1.13), P=0.10 | 1.16 (0.97-1.38), P=0.10 | 0.16 |
| All-cause mortality | |  |  |
| Observed ASVs | 0.78 (0.69-0.87), P<0.001 | 0.58 (0.42-0.80), P<0.001 | <0.001 |
| Faith's PD | 0.80 (0.71-0.91), P<0.001 | 0.60 (0.43-0.82), P=0.001 | <0.001 |
| Shannon–Weiner index | 0.78 (0.70-0.86), P<0.001 | 0.57 (0.41-0.77), P<0.001 | <0.001 |
| Simpson index | 0.84 (0.77-0.92), P<0.001 | 0.57 (0.41-0.78), P<0.001 | <0.001 |
| CVD mortality |  |  |  |
| Observed ASVs | 0.77 (0.64-0.93), P=0.01 | 0.61 (0.36-1.03), P=0.07 | 0.02 |
| Faith's PD | 0.83 (0.68-1.01), P=0.05 | 0.58 (0.35-0.97), P=0.04 | 0.02 |
| Shannon–Weiner index | 0.77 (0.66-0.91), P=0.002 | 0.62 (0.38-1.02), P=0.06 | 0.01 |
| Simpson index | 0.83 (0.72-0.96), P=0.01 | 0.59 (0.35-0.99), P=0.049 | 0.04 |

Table S17: The comparison of Fine–Gray proportional subdistribution hazards and CVD-specific COX models.

|  |  | Multivariable Cox | | | Fine-Gray | | |  |  |
| --- | --- | --- | --- | --- | --- | --- | --- | --- | --- |
| Metric |  | HR | 95% CI | P | SHR | 95% CI | P |  | SHR**-**HR |
| Observed ASVs | Per 1-SD | 0.81 | (0.68-0.96) | 0.016 | 0.83 | (0.69-0.99) | 0.037 |  | 0.02 |
| Observed ASVs | Q2 vs Q1 | 0.85 | (0.55-1.33) | 0.484 | 0.88 | (0.57-1.37) | 0.578 |  | 0.03 |
| Observed ASVs | Q3 vs Q1 | 0.58 | (0.35-0.94) | 0.029 | 0.60 | (0.36-1.00) | 0.049 |  | 0.02 |
| Observed ASVs | Q4 vs Q1 | 0.70 | (0.43-1.14) | 0.155 | 0.74 | (0.45-1.22) | 0.234 |  | 0.04 |
| Observed ASVs | P-trend |  |  | 0.062 |  |  | 0.116 |  |  |
| Faith's PD | Per 1-SD | 0.86 | (0.72-1.03) | 0.095 | 0.88 | (0.72-1.07) | 0.193 |  | 0.02 |
| Faith's PD | Q2 vs Q1 | 0.76 | (0.49-1.18) | 0.220 | 0.78 | (0.50-1.22) | 0.273 |  | 0.02 |
| Faith's PD | Q3 vs Q1 | 0.61 | (0.38-0.98) | 0.043 | 0.63 | (0.38-1.04) | 0.069 |  | 0.02 |
| Faith's PD | Q4 vs Q1 | 0.66 | (0.41-1.06) | 0.088 | 0.69 | (0.41-1.15) | 0.150 |  | 0.03 |
| Faith's PD | P-trend |  |  | 0.048 |  |  | 0.100 |  |  |
| Shannon–Weiner index | Per 1-SD | 0.79 | (0.68-0.92) | 0.003 | 0.81 | (0.69-0.95) | 0.010 |  | 0.02 |
| Shannon–Weiner index | Q2 vs Q1 | 0.82 | (0.53-1.26) | 0.369 | 0.83 | (0.54-1.27) | 0.389 |  | 0.01 |
| Shannon–Weiner index | Q3 vs Q1 | 0.57 | (0.35-0.93) | 0.024 | 0.58 | (0.36-0.95) | 0.029 |  | 0.01 |
| Shannon–Weiner index | Q4 vs Q1 | 0.62 | (0.39-1.00) | 0.048 | 0.65 | (0.41-1.06) | 0.082 |  | 0.03 |
| Shannon–Weiner index | P-trend |  |  | 0.018 |  |  | 0.036 |  |  |
| Simpson index | Per 1-SD | 0.82 | (0.72-0.93) | 0.003 | 0.83 | (0.74-0.93) | 0.001 |  | 0.01 |
| Simpson index | Q2 vs Q1 | 0.89 | (0.58-1.37) | 0.598 | 0.90 | (0.59-1.37) | 0.624 |  | 0.01 |
| Simpson index | Q3 vs Q1 | 0.80 | (0.51-1.26) | 0.332 | 0.82 | (0.52-1.28) | 0.384 |  | 0.02 |
| Simpson index | Q4 vs Q1 | 0.57 | (0.35-0.93) | 0.024 | 0.60 | (0.36-0.97) | 0.036 |  | 0.03 |
| Simpson index | P-trend |  |  | 0.024 |  |  | 0.033 |  |  |

Figure S1: Association Between Shannon–Weiner index, Simpson index, and advanced CKM. Forest map (A, B), RCS analysis (C, D), generalized additive model analysis (E, F).

Figure S2: Association Between Observed ASVs, Faith's PD and CVD mortality. Forest map (A, B), RCS analysis (C, D), generalized additive model analysis (E, F).
